# Diagnostic accuracy of neutrophil-to-lymphocyte and platelet-to-lymphocyte ratios in differentiating thyroid tumors: A systematic review and meta-analysis

**DOI:** 10.1371/journal.pone.0322382

**Published:** 2025-05-05

**Authors:** Bisrat Birke Teketelew, Dereje Mengesha Berta, Elias Chane, Amare Mekuanint, Tekletsadik Tekleslassie Alemayehu, Zewudu Mulatie, Muluken Walle, Abiy Ayele Angelo, Negesse Cherie

**Affiliations:** 1 Department of Hematology and Immunohematology, University of Gondar College of Medicine and Health Sciences, Gondar, Ethiopia; 2 Department of clinical chemistry, University of Gondar College of Medicine and Health Sciences, Gondar, Ethiopia; 3 Department of pharmacology, School of Biomedical and Laboratory Sciences, College of Medicine and Health Sciences, University of Gondar, Ethiopia; 4 Department of Hematology & Immunohematology, Wollo University, Ethiopia,; 5 Department of immunology and molecular biology, University of Gondar College of Medicine and Health Sciences, Gondar, Ethiopia; 6 Department of Quality assurance and laboratory management, University of Gondar College of Medicine and Health Sciences, Gondar, Ethiopia; University of Montenegro-Faculty of Medicine, MONTENEGRO

## Abstract

**Background:**

Thyroid neoplasms include a range of lesions, most of which are benign, though some may progress to or present as malignant. Diagnostic tools like FNAB, ultrasound, and hormone analysis are commonly used, though they have limitations. Recently, peripheral blood markers have been explored for their potential in differentiating thyroid lesions, despite controversy evidence. This review evaluates the diagnostic utility of NLR and PLR in thyroid lesions.

**Methods:**

We systematically searched all relevant articles on PubMed, Science Direct, Cochrane Library, and gray literature, including Google Scholar, for studies on the diagnostic utility of platelet-to-lymphocyte ratio (PLR) and neutrophil-to-lymphocyte ratio (NLR) in thyroid lesions. Two researchers independently screened articles, and study quality was assessed using the QUADAS 2 tool. A random-effects model calculated pooled sensitivity and specificity, while the area under the HSROC curve summarized diagnostic accuracy. Heterogeneity was evaluated with Higgins’ I² statistic, and publication bias was assessed using the MIDAS command. Subgroup analyses by sample size, gender distribution, cutoff values, and carcinoma types explored sources of heterogeneity.

**Results:**

A total of 12 studies were included in the final meta-analysis, with 9 focusing on NLR and 6 on PLR. Most of these studies were retrospective in design. The pooled sensitivity and specificity of NLR were 75% (95% CI: 65–82%) and 62% (95% CI: 42–75%), respectively. For PLR, the overall sensitivity and specificity were 70% (95% CI: 61–78%) and 57% (95% CI: 46–66%), respectively. The diagnostic accuracy, based on the area under the HSROC curve (AUC), was 0.75 (95% CI: 0.71–0.79) for NLR and 0.69 (95% CI: 0.65–0.73) for PLR. These results indicate that NLR has better diagnostic accuracy than PLR in distinguishing between benign and malignant thyroid lesions.

**Conclusion:**

While the NLR demonstrates better diagnostic utility than the PLR in distinguishing between benign and malignant thyroid lesions, its standalone diagnostic accuracy remains moderate. Therefore, we recommend using these markers as complementary tools alongside other standard diagnostic modalities until further studies provide more definitive evidence.

Thyroid neoplasm is a type of cancer which arises from the thyroid parenchymal cell and affects the thyroid gland. The disease encompasses a variety of lesion, including benign adenomas to malignant carcinomas [1]. The majority of thyroid glands are noncancerous and are mostly benign. In some contexts, thyroid adenomas may transform into carcinomas as the nonfunctional adenomas possess oncogene mutations [2]. Overall, 5% of thyroid nodules are malignant. Notably, approximately 20% of follicular adenomas have the potential to progress into follicular carcinomas [3]. The common thyroid malignancies include, papillary, follicular, medullary and anaplastic types, which varies based on the aggressiveness of the cancer, which are highly variable clinical features; some may be indolent and slow progressing while others may be highly aggressive tumors with a high mortality rate [4,5]. Globally, thyroid cancer cases are increasing over time. Approximately 18.3 million thyroid cases were reported with a high prevalence rate in China and United States [6].

The Preferred Reporting Items for Systematic Reviews and Meta-Analyses (PRISMA) guideline was used and performed for this systematic review and meta-analysis. This review was registered on PROSPERO with registration number of CRD42024559798.

## Background

Thyroid nodules have several diagnostic methods, including Fine Needle Aspiration Cytology (FNAC), neck ultrasound, and quantification of Thyroid Stimulating Hormone (TSH) level. In patients with thyroid nodules, FNA is an important and accurate diagnostic modality in distinguishing between benign and malignant thyroid nodules. Unfortunately, about 30% of the cases yield indeterminate results, leading to diagnostic challenges [7,8]. Ultrasound is the most sensitive diagnostic modality for thyroid nodules using several features to distinguish adenomas and carcinomas, and assess with a high accuracy for differentiation. However, it needs high personal skill, and performance, and creates interobserver variability, which is mainly dependent on the skill and judgement of sonographer [9,10]. Moreover, ultrasound is a tedious procedure that may reduce the efficiency in diagnosing thyroid nodules [10]. Serum TSH level has high sensitivity and specificity in diagnosing thyroid cancer, however, due to overlapping and hidden clinical feature, there may delayed and mis diagnosis in thyroid nodules [11]. These limitations and challenges highlight the need for supplementary diagnostic methods to enhance accuracy, and hence, it is important to perform multidisciplinary diagnostic methods. Performing and interpreting complete blood count (CBC) parameters such as neutrophil, monocyte, lymphocyte and platelet including their ratio is an important supplementary, cost effective and efficient procedure for patient with various cancer types.

Peripheral blood inflammatory biomarkers have emerged as valuable indicators of the host inflammatory response and are closely linked to cancer pathophysiology and progression. Their role in solid tumors, including thyroid carcinomas, highlights their potential as diagnostic and prognostic markers [12]. Among these biomarkers, neutrophils, lymphocytes, and platelets play crucial roles in tumor-related inflammation within the tumor microenvironment. Elevated neutrophil levels contribute to tumor growth and invasion by releasing inflammatory factors, while increased platelet counts assist tumor cells in evading immune surveillance. In contrast, higher lymphocyte infiltration within tumors reflects a strong anti-tumor immune response, and show an increase in their levels in peripheral blood [13]. Conversely, a decline in lymphocyte levels indicates immune suppression and a weakened innate immune defense against malignant tumors. Given their significant role in tumor progression and immune response, these inflammatory biomarkers provide a promising, non-invasive tool for cancer diagnosis, prognosis, and treatment monitoring [13, 14].

In recent years, peripheral inflammatory biomarkers derived from routine blood tests, such as neutrophil to lymphocyte ratio (NLR), and platelet to lymphocyte ratio (PLR) have gained attention as potential predictors of diagnosing in various malignancies [15]. These markers reflect the systemic inflammatory response, which plays a critical role in tumor progression and immune evasion including, thyroid cancer [13, 16]. Hence, distinguishing the malignant transformation of thyroid nodule is an important clinical process, because approximately 10–15% of the thyroid adenomas may transform to carcinomas depending on various risks [9]. Studies have suggested that NLR and PLR may serve as cost-effective, and accessible biomarkers for differentiating benign and malignant thyroid lesions [17–19]. However, the diagnostic performance of NLR and PLR in thyroid tumors remains inconsistent across individual studies, with varying cut-off values and results reported in the literature. A systematic review and meta-analysis are necessary to synthesize the available evidence, assess the diagnostic accuracy of these biomarkers, and determine their potential utility in clinical practice. There is no meta-analysis conducted to show the exact diagnostic utility of these markers in differentiating between benign and malignant thyroid tumor Therefore, we conduct this first meta-analysis to evaluate the sensitivity, specificity, and overall diagnostic value of NLR and PLR in distinguishing thyroid tumors, providing directions into their applicability as supplementary tools in thyroid cancer diagnosis.

## Methods and materials

### Search strategy

A systematic literature search using PubMed, Science direct, and Cochrane library was done up to November 11/2024 to obtain studies showing the NLR and PLR in thyroid adenoma and carcinoma. The search terms utilized during searching were as follows “((neutrophil to lymphocyte ratio) OR (platelet to lymphocyte ratio)) AND ((((((thyroid adenoma) OR (thyroid carcinoma)) OR (thyroid nodule)) OR (thyroid malignant nodule)) OR (thyroid benign nodule)) OR (thyroid cancer))”. Other published articles were also searched from gray literatures and reference lists to included studies which may not include in the electronic data base searching.

### Inclusion and exclusion criteria

We included studies revealed on the diagnostic value of NLR and PLR in patient with thyroid nodule including thyroid adenomas and carcinomas. Retrospective studies and prospective studies were included. Whereas, case reports, review articles, pilot studies, studies on animal model, letters, meeting records were filtered and excluded during searching and articles with insufficient data to obtain sensitivity and specificity were excluded from the study. Furthermore, articles lacking complete data, such as sensitivity and specificity values, were excluded, as their absence could impact our analysis. Given that our study pools sensitivity and specificity to determine the overall diagnostic accuracy, including such incomplete data could compromise the reliability of the summary findings.

### Study selection process

After carefully searching by the search term, articles were exported to EndNote x8 and the selection process was made on this software to manage search results and remove duplicates. Based on the inclusion criteria two reviewers had independently screened articles based on their title and abstract. These two researchers also screened the full text of articles weather it meets the inclusion criteria. In cases of disagreement, a third reviewer carefully reviewed the articles and resolved any conflicts between the two reviewers.

### Data extraction and quality assessment

After careful selection of the study, two authors independently assessed the eligibility of studies based on the aforementioned criteria and assessed methodological quality based on Quality Assessment foe Diagnostic Accuracy Studies 2 (QUADAS 2) quality assessment tool [20], which assess the quality of studies based on the four domain of risk bias and three domain of applicability concern. Two authors carefully extracted information from all eligible studies. Discrepancies of the findings from each study between the reviewers were resolved by consensus. From each study, information including first author name, year of publication, gender, mean age, country, types of thyroid carcinomas, sample size, sensitivity and specificity of NLR and PLR were extracted using Microsoft excel software.

### Statistical analysis

After extracting data, meta-analysis was performed to evaluate the diagnostic performance of NLR and PLR in patient with thyroid adenomas and thyroid carcinomas. The pooled sensitivity and specificity with corresponding forest plot was analyzed. Moreover, Hierarchical Summary Receiver Operator Characteristic (HSROC) curve with 95% confidence interval was generated using Meta-analytical Integration of Diagnostic test Accuracy Studies (MIDAS) command. This model allows for both fixed and random effects relating to threshold, accuracy and ascertain a summary point of different accuracy parameters. Spearman’s correlation coefficient was done to assess the threshold effect between sensitivity and specificity. Fagan’s nanogram plot was done to determine the relationship between prior probability, likelihood ratio, and posterior probability. Heterogeneity assessment was taken from visual examination of the forest plot, Summary Receiver Operator Characteristic (SROC) space and the I^2^ from the forest plot. Furthermore, subgroup analysis and meta regression was done to identify the source of heterogeneity among studies stratified by sample size, cut off value, gender distribution, and types of carcinomas. To check the presence of publication bias, the Deek’s funnel plot asymmetry test using MIDAS command was analyzed.

## Result

### Study selection

**Fig 1:**
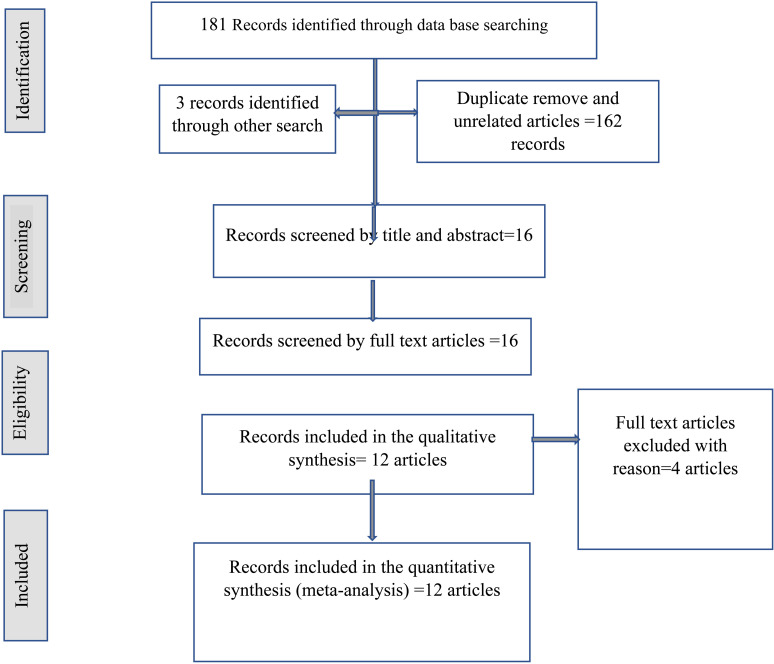
Flow chart of articles identified through literature search through data base and others.

The initial search strategy yielded 181 articles, of which 162 were excluded due to duplicate data or lack of relevance to the review’s objectives. After thoroughly reviewing the remaining 16 articles, an additional four articles were excluded because of incomplete data, leaving 12 studies for further analysis **(see** Fig 1**).** For the final meta-analysis, these 12 studies, encompassing a total of 4,141 subjects, were included. Among them, 9 studies [21–29], with a combined total of 2,886 subjects (2,106 with adenomas and 780 with thyroid carcinomas), primarily female (79.3%), met the inclusion criteria and provided complete diagnostic accuracy data for NLR. Additionally, six studies [22,23,29–32] with 2,193 subjects (1,497 with adenomas and 695 with thyroid carcinomas) were analyzed for the diagnostic accuracy of PLR **(see** Table 1**).**

**Table 1:** Characteristics of studies included in the final meta-analysis of the diagnostic accuracy of NLR and PLR in thyroid tumor.

Author, year	Mean age	Gender		Study design	Sample size			Sensitivity NLR	Specificity NLR	Sensitivity PLR	Specificity PLR
		Male	Female		Adenoma	Carcinoma	Total				
Mehmet Bug˘ra Bozan, 2020	50	49	194	retrospective cohort	184	50	234	62	49	–	–
Muzaffer Serdar Deniz, 2023	51.5 ± 13.5	92	367	retrospective cohort	438	21	459	62	75	71	71
Yuanyuan Deng, 2022	48	103	412	retrospective	374	140	514	–	–	54	62
Dimitrios K. Manatakis, 2018	53.01 ± 14.5	94	303	retrospective	190	207	397	80	34	73	37
Derya Kocer, 2015	50.41 ± 12.45	89	143	retrospective	167	65	232	89	54	–	–
Satriya Kelana, 2022	–	15	47	retrospective cohort	10	52	62	69	70	–	–
Hakan Bölükbaş, 2020	48.01 ± 12.23	105	508	retrospective	417	196	613	82	33	–	–
Burcin Meryem Atak Tel, 2021	43.85 ± 9.5	104	339	retrospective	207	236	443	–	–	69	51
Hayri Bostan, 2022	48.3 ± 12.3	25	175	retrospective	78	122	200	49	81	–	–
Mustafa C Şenoymak, 2024	53 medians	123	484	prospective cohort	573	34	607	82	83	–	–
Haider Salim Mihson, 2022	52.65 ± 16.26	15	67	cohort	49	33	82	85	71	88	71
Chiara Off, 2021	50.69 ± 14.73	63	235	retrospective	239	59	298	–	–	69	48

#### Quality assessment.

**Fig 2:**
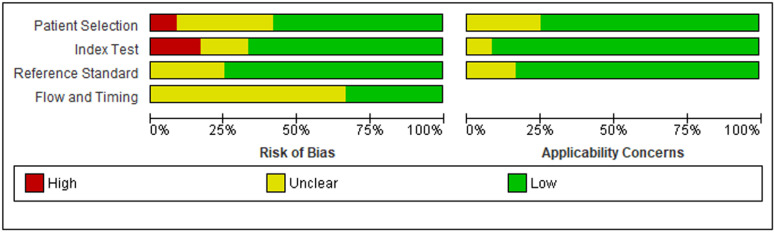
Graphical presentation of the quality of studies.

The quality assessment of included studies was done based on the QUADAS 2 criteria and properly outlined in Fig 2. Based on the assessment, 7 (58.3%), 8 (66.7%), 9 (75%) and 4(33.3%) of the studies were deemed to carry low risk of bias within the selection, index, reference standard and flow and timing domain, respectively. One study in the patient selection domain and two studies in the index test domain deemed to carry high risk of bias. Moving to the applicability concern, most of the studies meet the low applicability concern **(see** Figs 2 **and** 3**).**

**Fig 3:**
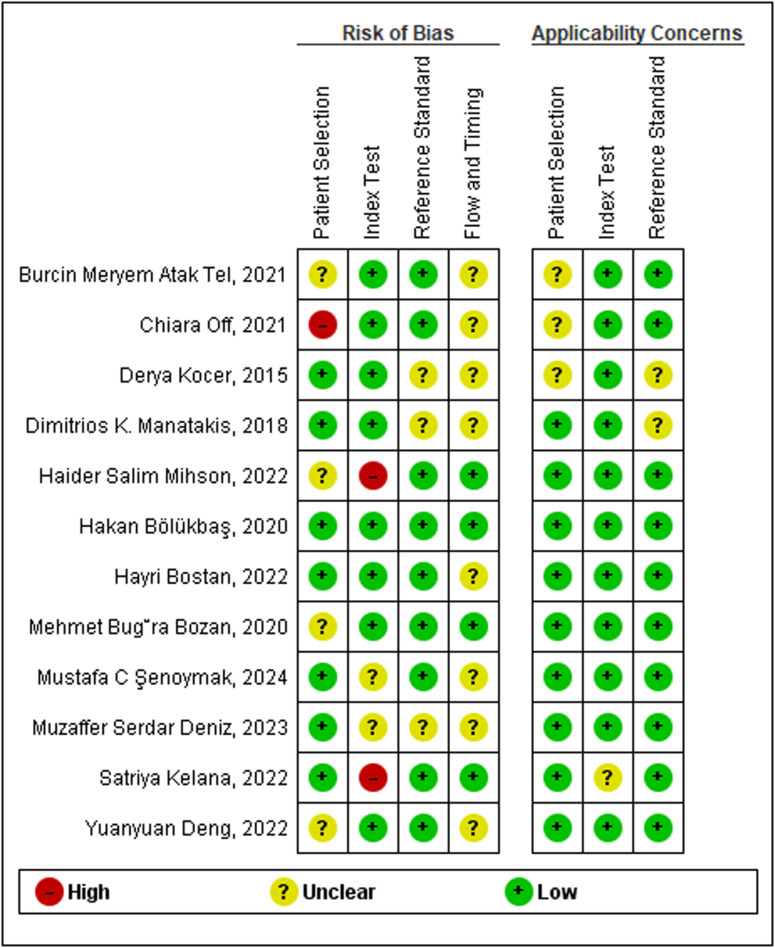
The figure presents the assessment of the risk of bias and applicability concerns for included studies based on QUADAS 2 tool. The “Risk of Bias” section evaluates four domains: Patient Selection, Index Test, Reference Standard, and Flow and Timing. The “Applicability Concerns” section assesses concerns related to Patient Selection, Index Test, and Reference Standard.

## Result

### Diagnostic accuracy of Neutrophil to lymphocyte ratio thyroid tumor.

**Fig 4:**
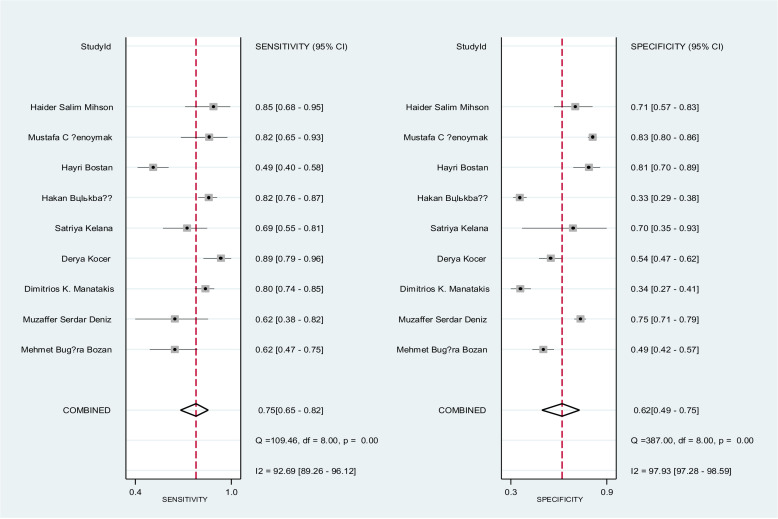
This figure presents forest plots for the sensitivity and specificity of nine studies evaluating a diagnostic accuracy of NLR. Each individual study is represented by a square, and the pooled sensitivity and specificity indicated by the diamond shape. The horizontal lines indicate the 95% confidence intervals (CIs).

**Fig 5:**
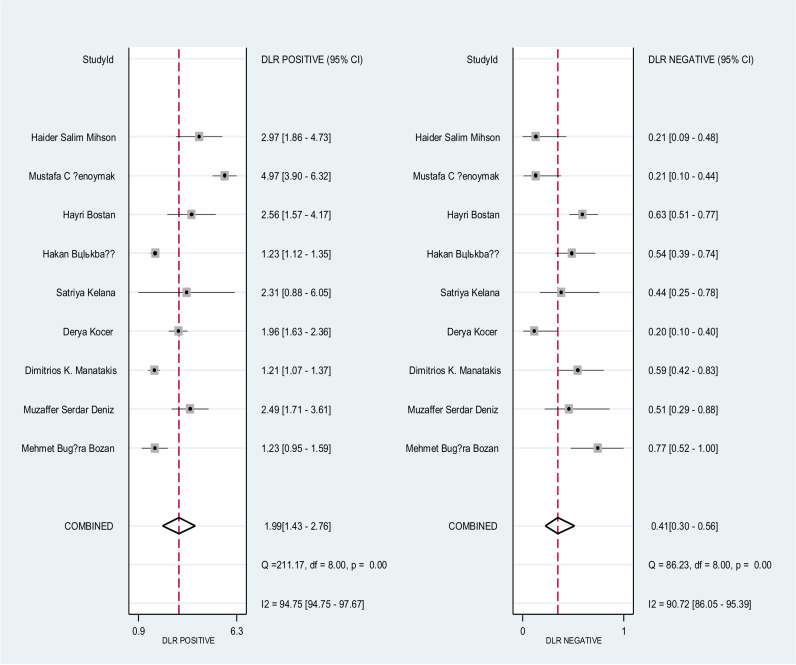
This figure presents forest plots for the diagnostic likelihood ratio (DLR) of various studies evaluating a diagnostic accuracy of NLR.

**Fig 6:**
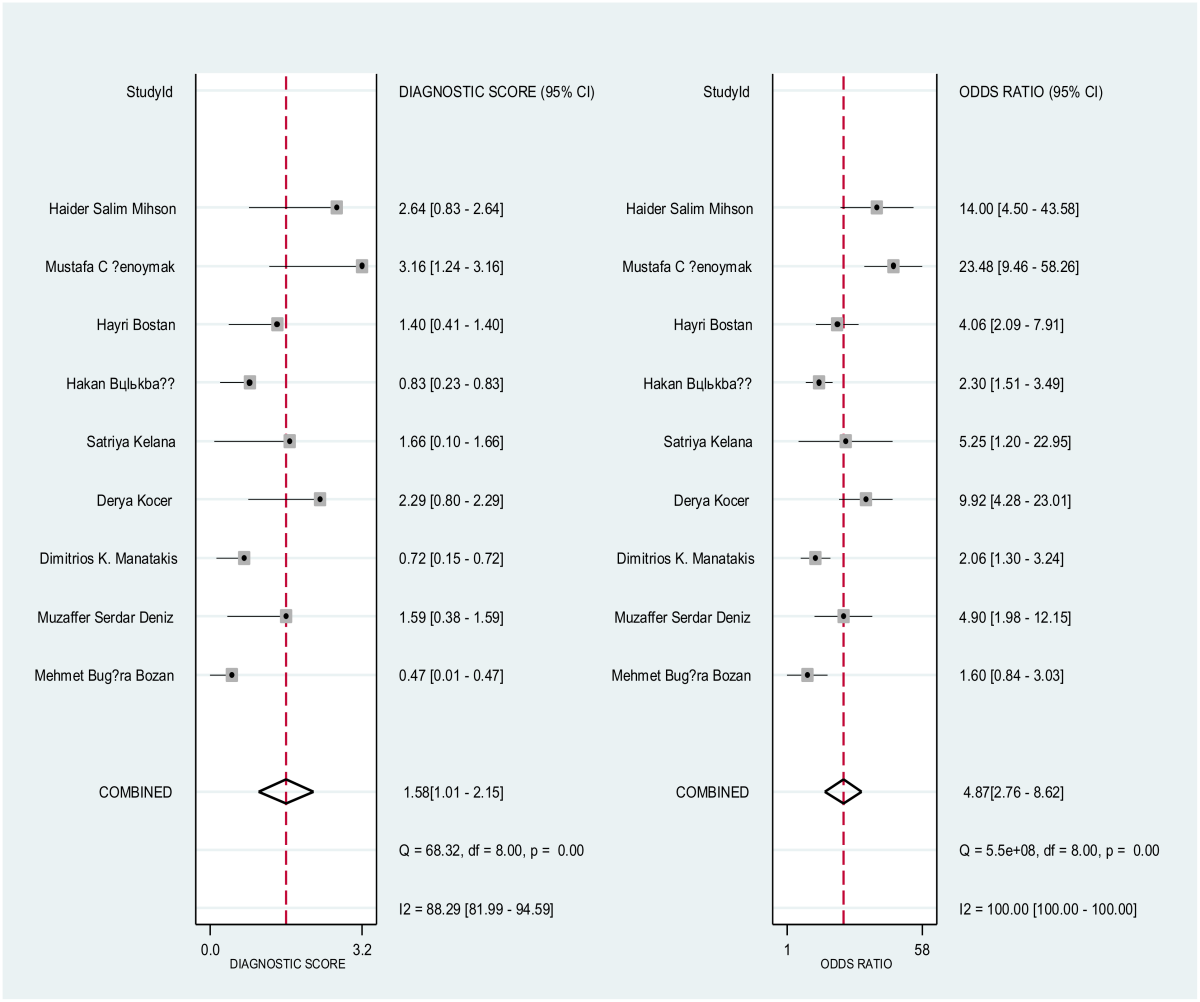
This figure presents forest plots for the diagnostic likelihood ratio (DLR) of various studies evaluating a diagnostic accuracy of NLR.

**Fig 7:**
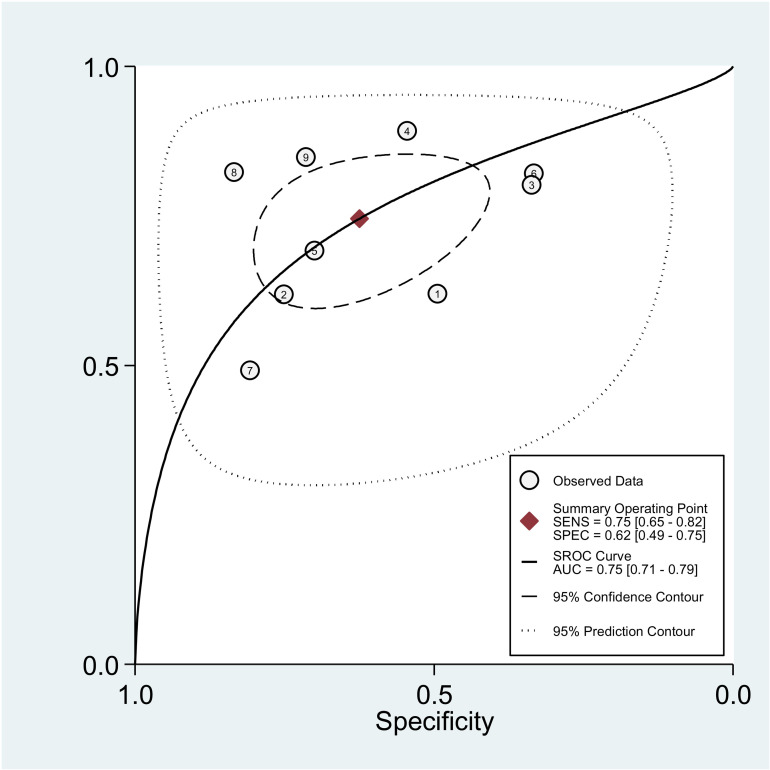
This figure displays the Summary Receiver Operating Characteristic (SROC) curve, which represents the diagnostic accuracy of NLR by combining sensitivity and specificity data from the nine included studies.

Forest plot on **Fig 4** shows the overall sensitivity and specificity of NLR for the nine included studies. The pooled sensitivity and specificity across all studies was 75% (95% CI: 65–82%) and 62% (95% CI: 42–75%), respectively. The pooled estimates of DOR for NLR was 4.87 (95% CI: 2.78–8.62) **Fig 5**, and the pooled positive and negative likelihood ratio of NLR was 1.99 (95 CI: 1.43–2.76) and 0.41 (95% CI: 0.30–0.56), respectively, **Fig 6**. In **Fig 7**, the HSROC curve together with the summary point of sensitivity and specificity with 95% CI was presented. Based on the analysis, the area under the HSROC curve was 0.75 (95 CI: 0.71–0.79). We observed a notable heterogeneity among the included studies, suggesting that there was a wide variation in sensitivity and specificity between the studies.

### Fagan test for NLR.

Fagan’s nomogram showed that, assuming a pre-test probability of thyroid carcinoma of 20%, the post-test probability was 35% in subjects with relatively high NLR values and 10% in those with relatively low NLR values

### Subgroup analysis for NLR

presents a meta-analysis of subgroup on pooled sensitivity and specificity of NLR for various factors. Studies with a sample size ≤ 200 showed higher variation in sensitivity compared to those with > 200. A cut-off value of NLR < 2 also yielded higher sensitivity but significantly lower specificity. Male-to-female ratios and carcinoma types showed minor variations in sensitivity and specificity, with studies examining multiple carcinoma types generally exhibiting better diagnostic performance. Significant subgroup differences were observed in several categories, particularly for cut-off values and carcinoma types.

**Table 2:** Subgroup analysis to check the source of heterogeneity for NLR (n = 9).

Subgroup	Category	Number of studies	Pooled sensitivity	I^2^	Subgroup difference p-value	Pooled specificity	I^2^	Subgroup difference p-value
Sample size	>200	6	0.78 [0.70 - 0.86]	25	0.68	0.56 [0.42 - 0.71]	0	0.09
	<=200	3	0.67 [0.52 - 0.82]		0.02*	0.75 [0.57 - 0.93]		0.49
Cut off value	>=2	5	0.70 [0.58 - 0.82]	87	0.01*	0.78 [0.72 - 0.84]	73	0.01*
	<2	4	0.80 [0.71 - 0.89]		0.52	0.42 [0.34 - 0.50]		<0.001*
Male to female ratio	>=1:4	6	0.76 [0.66 - 0.85]	0	0.31	0.62 [0.46 - 0.79]		0.73
	<1:4	3	0.73 [0.58 - 0.87	0	0.14	0.63 [0.40 - 0.86]	0	0.76
Types of carcinoma	Single type	4	0.70 [0.58 - 0.83]	30	0.03*	0.57 [0.37 - 0.77]		0.31
	Two and more type	5	0.78 [0.68 - 0.88]		0.47	0.67 [0.51 - 0.84]	0	0.83

### Publication bias

**Fig 8:**
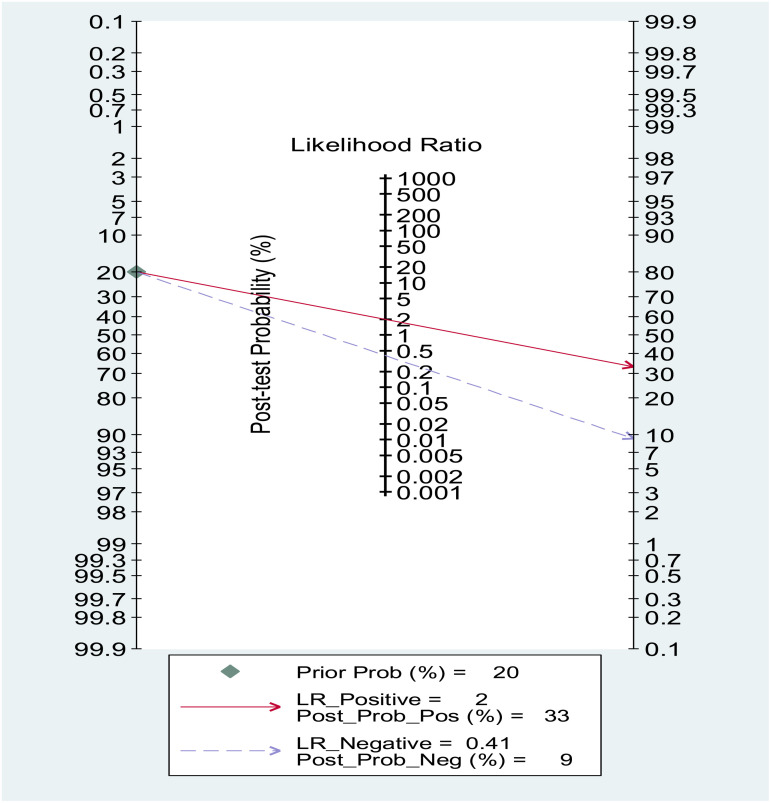
Illustrates a Fagan nomogram, a graphical tool used to determine the post-test probability of a condition based on the pre-test probability and the diagnostic test’s likelihood ratios.

The Deek’s funnel plot asymmetry test shows no significant asymmetry, with a p-value of 0.10, indicating no evidence of publication bias. The diagnostic odds ratios (x-axis) appear evenly distributed across the range of 1/root (ESS) (y-axis). This suggests the diagnostic accuracy results are unlikely to be influenced by small-study effects (**seen** Fig 8).

#### Diagnostic accuracy of platelet to lymphocyte ratio between benign and malignant thyroid tumor.

**Fig 9:**
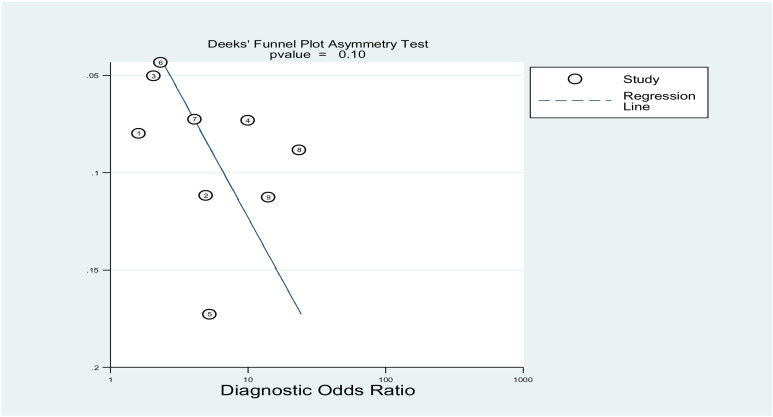
The Deek’s funnel plot asymmetry test was used in our review to assess publication bias. The analysis showed that the diagnostic odds ratio was symmetrically distributed across the range of sample sizes, indicating that publication bias was very unlikely.

**Fig 10:**
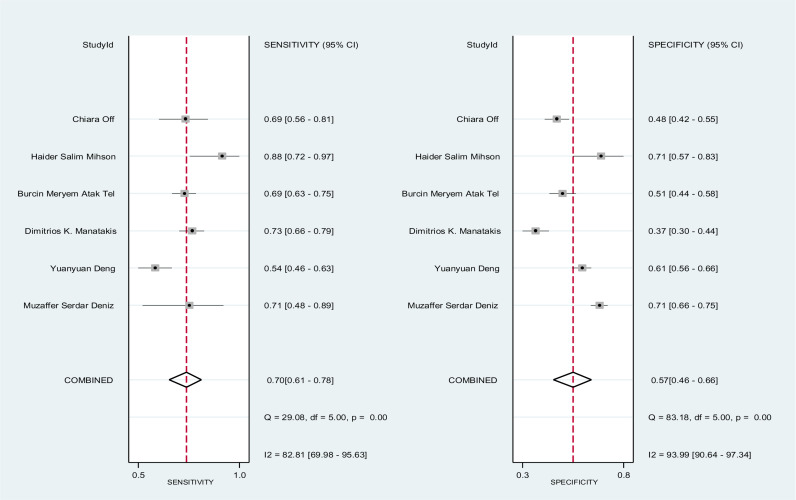
The figure presents forest plots for the sensitivity and specificity of six studies evaluating a diagnostic accuracy of PLR. Each individual study is represented by a square, and the pooled sensitivity and specificity indicated by the diamond shape. The horizontal lines indicate the 95% confidence intervals (CIs).

**Fig 11:**
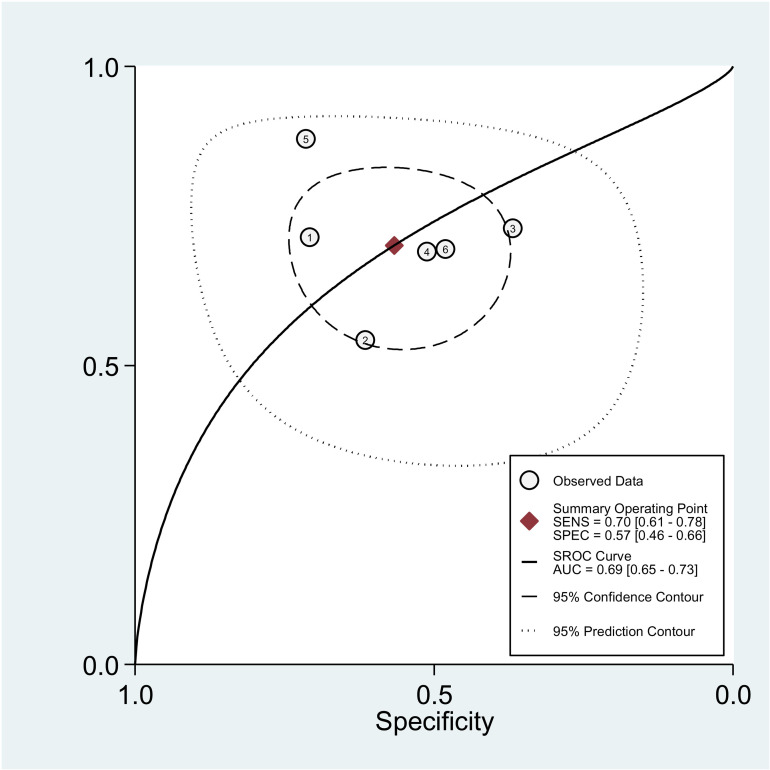
This figure displays the Summary Receiver Operating Characteristic (SROC) curve, which represents the diagnostic accuracy of PLR by combining sensitivity and specificity data from the six included studies.

**Fig 12:**
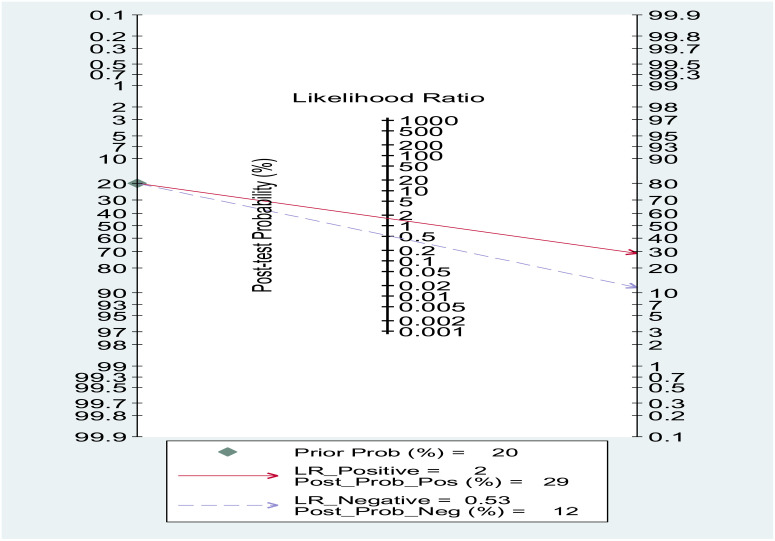
Illustrates a Fagan nomogram, a graphical tool used to determine the post-test probability of a condition based on the pre-test probability and the diagnostic test’s likelihood ratios.

The diagnostic accuracy of PLR was evaluated across six studies, as shown in Fig 4. The pooled sensitivity and specificity were 70% (95% CI: 61–78%) and 57% (95% CI: 46–66%), respectively. The pooled DOR for PLR was estimated at 3.06 (95% CI: 1.70–5.53). Additionally, the pooled positive and negative likelihood ratios for NLR were 1.62 (95% CI: 1.23–2.12) and 0.53 (95% CI: 0.38–0.74), respectively, as illustrated in **Fig 9**. **Fig 10** presents the HSROC curve, including the summary sensitivity and specificity point with a 95% CI. The area under the HSROC curve was 0.69 (95% CI: 0.65–0.73), indicating sufficient level of accuracy [33]. A high level of heterogeneity on the specificity of the studies was observed (I² = 93%) among the studies, indicating substantial variability in sensitivity and specificity across the included studies in **Fig 11**. Fig 12.

### Subgroup analysis for PLR

A subgroup analysis was conducted on pooled sensitivity and specificity of PLR for various factors. Studies with varied sample size showed higher variation in sensitivity compared to those without affecting specificity. Studies with lower cut-off value yielded lower sensitivity and lower specificity. Male-to-female ratios and carcinoma types showed no significant variation in sensitivity and specificity **(see** Table 3**).**

**Table 3:** Subgroup analysis to check the source of heterogeneity for PLR (n = 6).

Subgroup	Category	Number of studies	Pooled sensitivity	I^2^	Subgroup difference p-value	Pooled specificity	I^2^	Subgroup difference p-value
Sample size	>200	5	0.65 [0.58 - 0.72]	79	0.01*	0.54 [0.44 - 0.64]	55	0.15
	<=200	1	0.88 [0.76 - 1.00]		0.01*	0.72 [0.50 - 0.93]		0.52
Cut off value	>=140	2	0.81 [0.70 - 0.93]	87	0.86	0.70 [0.60 - 0.80]	73	0.37
	<140	4	0.67 [0.60 - 0.73]		<0.001*	0.50 [0.42 - 0.57]		<0.001*
Male to female ratio	>=1:4	4	0.72 [0.63 - 0.81]	14	0.42	0.52 [0.41 - 0.63]	0	0.11
	<1:4	2	0.67 [0.52 - 0.82]		0.12	0.67 [0.52 - 0.82]		0.63
Types of carcinoma	Single type	3	0.72 [0.62 - 0.83]	59	0.28	0.53 [0.39 - 0.66]	0	0.31
	Two and more type	3	0.68 [0.58 - 0.79]		0.07	0.61 [0.47 - 0.75]		0.83

### Fagan test for PLR

Fagan’s nomogram showed that, assuming a pre-test probability of thyroid carcinoma of 20%, the post-test probability was 30% in subjects with relatively high PLR values and 11% in those with relatively low PLR values

## Discussion

Thyroid nodules have become increasingly prevalent, with an estimated prevalence of 29.9% by the end of 2022 [34]. The malignancy rate among thyroid nodules was reported at 11% [35]. This rise in the prevalence of thyroid malignancies can be attributed to the widespread use of ultrasound for diagnosing thyroid nodules. Consequently, multiple diagnostic approaches are necessary to identify clinically significant thyroid nodules while avoiding overdiagnosis and overtreatment [36]. Currently, diagnostic methods such as FNAB, ultrasound, and innovative techniques like point-of-care (POC) thyroglobulin testing and liquid biopsy are commonly used to detect thyroid carcinomas [37]. However, these methods are often invasive, expensive, require specialized expertise, and may not be readily available in many healthcare systems, particularly in developing countries. As a result, researchers have turned their attention to simpler, more accessible, and cost-effective diagnostic tools, such as NLR and PLR. These parameters have shown potential for differentiating between benign and malignant thyroid nodules, although the results have been inconsistent. This has motivated us to conduct a meta-analysis to evaluate the exact diagnostic utility of NLR and PLR by analyzing pooled sensitivity, specificity, and summary points under HSROC.

This meta-analysis provides a comprehensive evaluation of the diagnostic accuracy of the NLR and PLR in distinguishing between benign and malignant thyroid nodules. The pooled data from twelve studies, nine of them assessing NLR and six studies assessing PLR, demonstrate moderate accuracy in identifying malignancy among thyroid tumor patients [33]. Specifically, NLR showed a pooled sensitivity of 75% and specificity of 62%, with an area under the HSROC curve of 0.75, suggesting a fair level of diagnostic accuracy. PLR demonstrated a pooled sensitivity of 70% and specificity of 57%, with an area under the HSROC curve of 0.69, indicating limited diagnostic accuracy. However, notable heterogeneity across studies was observed, underscoring the variability in test performance and the need for further research to refine and validate these markers in clinical settings.

This review findings suggest that NLR may offer slightly better diagnostic accuracy than PLR in differentiating malignant from benign thyroid lesions, with higher sensitivity and a better area under the HSROC curve, which suggests it may be a more reliable indicator for malignancy in thyroid assessments. The greater diagnostic accuracy performance of NRL could be attributed to its closer reflection of the inflammatory response during malignant condition [38]. Inflammatory processes play a crucial role in tumorigenesis, promoting cellular changes and supporting a microenvironment conducive to cancer progression [39]. As such, NLR might reflect the inflammatory situation linked with malignant transformation more accurately than PLR, which could explain its higher sensitivity in identifying malignancies in thyroid nodule [40]. However, both markers have limited specificity, meaning they should be used in conjunction with other diagnostic tools, such as ultrasound or fine-needle aspiration biopsy (FNAB), rather than as standalone markers.

Our heterogeneity analysis indicated substantial variability in sensitivity and specificity across the studies included in this review with overall heterogeneity for NLR (I^2^ = 98%), and for PLR (I^2^ = 93.99%). To investigate the sources of this heterogeneity, we conducted a subgroup analysis separately for NLR and PLR based on several key factors: sample size, cut-off values, gender distribution (male-to-female ratio), and types of carcinomas. Based on the subgroup analysis, variations in cut-off values for NLR and PLR, which were not consistent across studies, could contribute to the inconsistency in results, especially, for studies utilizing higher cut off value for NLR contributed for the significant variation both in sensitivity and specificity. On the other hand, studies utilizing lower cut off for PLR contributed for the high variability for sensitivity and specificity. Additionally, our review demonstrated that variation in sample size contributed to the variability of sensitivity for both NLR and PLR, particularly studies with smaller sample. This variability due to sample size variation might be attributed to the higher chance to being influenced by the random variation and imprecision of sensitivity when using smaller sample size [41].

Studies focusing on a single type of carcinoma have demonstrated significant variability in the sensitivity of diagnosing thyroid carcinoma. This variability arises because the sensitivity of a diagnostic test tends to fluctuate when it is applied to a single disease [42], even while maintaining a consistent level of specificity. This phenomenon highlights the inherent challenges in achieving uniformly high sensitivity across different cases within the same disease category, potentially due to biological heterogeneity, variations in disease presentation, or differences in the test’s detection mechanisms [43]. Moreover, studies conducting on a single type of carcinoma shows a significant variability in sensitivity. This is due to the fact that the sensitivity of a diagnostic test is variable when conducting on a single disease while maintaining specificity. However, gender distribution did not significantly affect the pooled sensitivity and specificity. Additionally, while we included studies on various carcinoma types, these differences did not significantly impact on the specificity. But, more prospective studies should be needed to assess the real diagnostic utility. Overall, the heterogeneity in results seems to stem primarily from inconsistencies in the cut-off values, using smaller sample size and conducting on a single type of carcinoma contributed for the significant variability in the diagnostic sensitivity of NLR and PLR.

Although NLR exhibit moderate diagnostic accuracy for differentiating benign and malignant thyroid nodule, the relatively low specificity and high variability across studies may limit the clinical utility as a definitive diagnostic marker for the condition. These parameters could, however, serve as screening and adjunctive tools with FNA in differentiating between benign and malignant thyroid nodule, and may helping to identify patients who benefits from invasive diagnostic procedure. Moreover, NLR, with its higher diagnostic accuracy compared to PLR, may be useful for risk stratification in patients with thyroid nodules. Patients with elevated NLR could be prioritized for FNAB or more intensive follow-up, thereby optimizing resource allocation. The link between NLR and inflammation in cancer provides useful information about how tumors develop. It can also help doctors identify more aggressive cancers and choose the best treatment plans. Nevertheless, the current evidence is insufficient to recommend NLR or PLR as reliable biomarkers in clinical practice. This review has both strengths and limitations. Its strengths include a comprehensive evaluation of multiple studies, a direct comparison of NLR and PLR, and the use of HSROC curves, which offer a robust assessment of diagnostic accuracy by accounting for variations in sensitivity and specificity across studies. However, the review has some limitations, including small sample sizes in the included studies, restricted subgroup analysis due to missing data, and limited geographic diversity, as most studies were conducted in specific regions. These factors may limit the generalizability of the findings to populations with different demographic and clinical characteristics.

## Conclusion

This meta-analysis provides a comprehensive assessment of the diagnostic utility of NLR and PLR in distinguishing between benign and malignant thyroid nodules. While NLR demonstrated slightly higher diagnostic accuracy compared to PLR, with a sensitivity of 75%, specificity of 62%, and an area under the HSROC curve of 0.75, both markers exhibited limited specificity and significant heterogeneity across studies. NLR, reflecting inflammatory processes associated with malignant transformation, shows promise as a supportive diagnostic tool, especially in combination with established methods like ultrasound or FNAB. However, PLR demonstrates potential but is less reliable as a diagnostic marker due to lower sensitivity and specificity. Nevertheless, its limited specificity and variability across studies underscore the need for further research to refine its clinical applicability. Expanding studies to include multicenter trials with larger and more diverse populations could also improve the generalizability of findings and refine the clinical application of NLR and PLR in thyroid cancer diagnostics. Moreover, we recommend further studies to explore the integration of NLR and PLR with ultrasound and FNAB to enhance diagnostic accuracy. Large-scale, prospective research is needed to assess their combined utility and establish standardized cut-off values for better clinical application.

## Supporting information

S1 File Prisma checklist. (DOCX)

S2 File QUADAS 2 tool and confirmation for eligibility. (DOCX)

S3 File List of excluded articles. (DOCX)

S4 File Summary of all extracted dada. (DOCX)

S5 File List of included articles in the final meta-analysis. (DOCX)
