# Supplementary material for: Diagnostic accuracy of neutrophil-to-lymphocyte and platelet-to-lymphocyte ratios in differentiating thyroid tumors: A systematic review and meta-analysis
Source: PLoS One. 2025 May 5;20(5):e0322382. doi: 10.1371/journal.pone.0322382 (PMC12052148; doi:10.1371/journal.pone.0322382)
Supplement: S2 File — (DOCX) [file pone.0322382.s002.docx]

**Table: Quality assessment for the included studies based on the QUADAS2 tool and confirmation for eligibility**

| S/N | Study | Risk of bias | | | | Applicably | | | **Confirmation for eligibility** |
| --- | --- | --- | --- | --- | --- | --- | --- | --- | --- |
|  |  | **Patient Selection** | **Index Test** | **Reference Standard** | **Flow & Timing** | **Patient Selection** | **Index Test** | **Reference Standard** |  |
| 1 | Mehmet Bug˘ra Bozan, 2020 | unclear | Low | Low | Low | Low | Low | Low |  |
| 2 | Muzaffer Serdar Deniz, 2023 | Low | Unclear | Unclear | Unclear | Low | Low | Low |  |
| 3 | Yuanyuan Deng, 2022 | Unclear | Low | Low | unclear | Low | Low | Low |  |
| 4 | Dimitrios K. Manatakis, 2018 | Low | Low | Unclear | Unclear | Low | Low | Unclear |  |
| 5 | Derya Kocer, 2015 | Low | Low | Unclear | Unclear | Unclear | Low | Unclear |  |
| 6 | Satriya Kelana, 2022 | Low | High | Low | Low | Low | Unclear | Low |  |
| 7 | Hakan Bölükbaş, 2020 | Low | Low | Low | Low | Low | Low | Low |  |
| 8 | Burcin Meryem Atak Tel, 2021 | Unclear | Low | Low | Unclear | Unclear | Low | Low |  |
| 9 | Hayri Bostan, 2022 | Low | Low | Low | Unclear | Low | Low | Low |  |
| 10 | Mustafa C Şenoymak, 2024 | Low | Unclear | Low | Unclear | Low | Low | Low |  |
| 11 | Haider Salim Mihson, 2022 | Unclear | High | Low | Low | Low | Low | Low |  |
| 12 | Chiara Off, 2021 | High* | Low | Low | Unclear | Unclear | Low | Low |  |
