# Supplementary material for: Diagnostic accuracy of neutrophil-to-lymphocyte and platelet-to-lymphocyte ratios in differentiating thyroid tumors: A systematic review and meta-analysis
Source: PLoS One. 2025 May 5;20(5):e0322382. doi: 10.1371/journal.pone.0322382 (PMC12052148; doi:10.1371/journal.pone.0322382)
Supplement: S3 File — (DOCX) [file pone.0322382.s003.docx]

**Table: List of excluded articles from the total search and not included in the final meta-analysis (N=169)**

| **S/N** | Author year | **Articles title** | **Reason for exclusion** |
| --- | --- | --- | --- |
| 1 | [Rosaria Maddalena Ruggeri](https://pubmed.ncbi.nlm.nih.gov/?term=Ruggeri+RM&cauthor_id=32729374), 2020 | Influence of Dietary Habits on Oxidative Stress Markers in Hashimoto's Thyroiditis | Not related to the review objective and excluded by title and abstract |
| 2 | [Yiqun Hu](https://pubmed.ncbi.nlm.nih.gov/?term=Hu+Y&cauthor_id=36343549) , 2023 | Alantolactone induces concurrent apoptosis and GSDME-dependent pyroptosis of anaplastic thyroid cancer through ROS mitochondria-dependent caspase pathway | Not related to the review objective and excluded by title and abstract |
| 3 | [Jingting Wang](https://pubmed.ncbi.nlm.nih.gov/?term=Wang+J&cauthor_id=38267897), 2024 | Predictive biomarkers for immune-related adverse events in cancer patients treated with immune-checkpoint inhibitors | Not related to the review objective and excluded by title and abstract |
| 4 | [Patrick A Ott](https://pubmed.ncbi.nlm.nih.gov/?term=Ott+PA&cauthor_id=30557521) | T-Cell-Inflamed Gene-Expression Profile, Programmed Death Ligand 1 Expression, and Tumor Mutational Burden Predict Efficacy in Patients Treated With Pembrolizumab Across 20 Cancers: KEYNOTE-028 | Not related to the review objective and excluded by title and abstract |
| 5 | [Alessandro Antonelli](https://pubmed.ncbi.nlm.nih.gov/?term=Antonelli+A&cauthor_id=26521772) | Editorial: New Therapies, Markers and Therapeutic Targets in HCV Chronic Infection and HCV Extrahepatic Manifestations | Not related to the review objective and excluded by title and abstract |
| 6 | [Rasoul Yahyapour](https://pubmed.ncbi.nlm.nih.gov/?term=Yahyapour+R&cauthor_id=29554942), 2018 | Radiation-induced inflammation and autoimmune diseases | Not related to the review objective and excluded by title and abstract |
| 7 | [Mehmet Sercan Erturk](https://pubmed.ncbi.nlm.nih.gov/?term=Erturk+MS&cauthor_id=32811597), 2020 | Microwave Ablation of Benign Thyroid Nodules: Effects on Systemic Inflammatory Response | Not related to the review objective and excluded by title and abstract |
| 8 | [Sorina Martin](https://pubmed.ncbi.nlm.nih.gov/?term=Martin+S&cauthor_id=34829306) | Platelet Activation and Inflammation in Patients with Papillary Thyroid Cancer | Not related to the review objective and excluded by title and abstract |
| 9 | [Claudio Gambardella](https://pubmed.ncbi.nlm.nih.gov/?term=Gambardella+C&cauthor_id=37047439), 2023 | Role of Inflammatory Biomarkers (NLR, LMR, PLR) in the Prognostication of Malignancy in Indeterminate Thyroid Nodules | Not related to the review objective and excluded by title and abstract |
| 10 | [Mitsuo Yokota](https://pubmed.ncbi.nlm.nih.gov/?term=Yokota+M&cauthor_id=31711613)^,^ 2020 | Lymphocyte-Monocyte Ratio Significantly Predicts Recurrence in Papillary Thyroid Cancer | Not related to the review objective and excluded by title and abstract |
| 11 | [Naoki Fukuda](https://pubmed.ncbi.nlm.nih.gov/?term=Fukuda+N&cauthor_id=32871825), 2020 | Neutrophil-to-Lymphocyte Ratio as a Prognostic Marker for Anaplastic Thyroid Cancer Treated With Lenvatinib | Not related to the review objective and excluded by title and abstract |
| 12 | [Katarzyna Cieplińska](https://pubmed.ncbi.nlm.nih.gov/?term=Ciepli%C5%84ska+K&cauthor_id=38202079), 2023 | Immunological Processes in the Orbit and Indications for Current and Potential Drug Targets | Not related to the review objective and excluded by title and abstract |
| 13 | [Hongxi Wang](https://pubmed.ncbi.nlm.nih.gov/?term=Wang+H&cauthor_id=38589799)^,^ 2024 | Development and validation of prediction models for papillary thyroid cancer structural recurrence using machine learning approaches | Not related to the review objective and excluded by title and abstract |
| 14 | [Nianhua Ding](https://pubmed.ncbi.nlm.nih.gov/?term=Ding+N&cauthor_id=38212845), 2024 | Prognostic value of baseline neutrophil/lymphocyte ratio in HER2-positive metastatic breast cancer: exploratory analysis of data from the CLEOPATRA trial | Not related to the review objective and excluded by title and abstract |
| 15 | [Lingyun Zhang](https://pubmed.ncbi.nlm.nih.gov/?term=Zhang+L&cauthor_id=32596828), 2020 | Diagnostic and prognostic value of preoperative systemic inflammatory markers in anaplastic thyroid cancer | Not related to the review objective and excluded by title and abstract |
| 16 | [Yuan-Yuan Guo](https://pubmed.ncbi.nlm.nih.gov/?term=Guo+YY&cauthor_id=36187616), 2022 | Machine learning for identifying benign and malignant of thyroid tumors: A retrospective study of 2,423 patients | Not related to the review objective and excluded by title and abstract |
| 17 | [Guoqiang Wang](https://pubmed.ncbi.nlm.nih.gov/?term=Wang+G&cauthor_id=37243870), 2023 | Predictive value of systemic inflammatory markers for recurrence of papillary thyroid cancer | Not related to the review objective and excluded by title and abstract |
| 18 | [Canxiao Li](https://pubmed.ncbi.nlm.nih.gov/?term=Li+C&cauthor_id=36060974)^,^ 2022 | Prognostic significance of inflammatory markers LMR, PLR, MPV, FIB in intermediate-and high-risk papillary thyroid carcinoma | Not report outcome variables |
| 19 | [N Altintas](https://pubmed.ncbi.nlm.nih.gov/?term=Altintas+N&cauthor_id=26400528), 2015 | Neutrophil-to-lymphocyte ratio in obstructive sleep apnea; a multi center, retrospective study | Not related to the review objective and excluded by title and abstract |
| 20 | [Marra Jai Aghajani](https://pubmed.ncbi.nlm.nih.gov/?term=Aghajani+MJ&cauthor_id=33112841) , 2020 | Epithelial-to-mesenchymal transition and its association with PD-L1 and CD8 in thyroid cancer | Not related to the review objective and excluded by title and abstract |
| 21 | [Dehui Qiao](https://pubmed.ncbi.nlm.nih.gov/?term=Qiao+D&cauthor_id=38568295) , 2024 | Nomogram to predict central lymph node metastasis in papillary thyroid carcinoma | Not related to the review objective and excluded by title and abstract |
| 22 | [Hyunjae Lee](https://pubmed.ncbi.nlm.nih.gov/?term=Lee+H&cauthor_id=37909732), 2023 | Value of circulating neutrophil elastase for detecting recurrence of differentiated thyroid cancer | Not report outcome variables |
| 23 | [Jiyun Park](https://pubmed.ncbi.nlm.nih.gov/?term=Park+J&cauthor_id=33921107), 2021 | Prognostic Value of the Neutrophil-to-Lymphocyte Ratio before and after Radiotherapy for Anaplastic Thyroid Carcinoma | Not related to the review objective and excluded by title and abstract |
| 24 | [Ashank Bains](https://pubmed.ncbi.nlm.nih.gov/?term=Bains+A&cauthor_id=34298730), 2021 | The Role of Vitamin D as a Prognostic Marker in Papillary Thyroid Cancer | Not related to the review objective and excluded by title and abstract |
| 25 | [Natália Medeiros Dias Lopes](https://pubmed.ncbi.nlm.nih.gov/?term=Lopes+NMD&cauthor_id=35902455) ,2022 | Role of papillary thyroid carcinoma patients with Hashimoto thyroiditis: evaluation of oxidative stress and inflammatory markers | Not related to the review objective and excluded by title and abstract |
| 162 | [Selahattin Vural](https://pubmed.ncbi.nlm.nih.gov/?term=Vural+S&cauthor_id=37543770), 2023 | Systemic immune-inflammation index: A new marker in differentiation of different thyroid diseases | Duplicate removed |
| 27 | [Qi Qi](https://pubmed.ncbi.nlm.nih.gov/?term=Qi+Q&cauthor_id=36251517), 2023 | Per- and polyfluoroalkyl substances activate UPR pathway, induce steatosis and fibrosis in liver cells | Not related to the review objective and excluded by title and abstract |
| 28 | [Lingqian Zhao](https://pubmed.ncbi.nlm.nih.gov/?term=Zhao+L&cauthor_id=38189045) | Preoperative risk stratification for patients with ≤ 1 cm papillary thyroid carcinomas based on preoperative blood inflammatory markers: construction of a dynamic predictive model | Not related to the review objective and excluded by title and abstract |
| 29 | [Selahattin Vural](https://pubmed.ncbi.nlm.nih.gov/?term=Vural+S&cauthor_id=37543770), 2023 | Systemic immune-inflammation index: A new marker in differentiation of different thyroid diseases | Not related to the review objective and excluded by title and abstract |
| 30 | [Arunangshu Ghoshal](https://pubmed.ncbi.nlm.nih.gov/?term=Ghoshal+A&cauthor_id=29416653) , 2017 | Thyroid cancer risk in the Swedish AMORIS study: the role of inflammatory biomarkers in serum | Not related to the review objective and excluded by title and abstract |
| 31 | [F De Paoli](https://pubmed.ncbi.nlm.nih.gov/?term=De+Paoli+F&cauthor_id=25941992), 2015 | The neuron-derived orphan receptor 1 (NOR1) is induced upon human alternative macrophage polarization and stimulates the expression of markers of the M2 phenotype | Not related to the review objective and excluded by title and abstract |
| 32 | [Ana Abrantes Figueiredo](https://pubmed.ncbi.nlm.nih.gov/?term=Figueiredo+AA&cauthor_id=36764748) , 2023 | Preoperative serum inflammation-based scores in medullary thyroid cancer | Not related to the review objective and excluded by title and abstract |
| 33 | [Haichuan Liu](https://pubmed.ncbi.nlm.nih.gov/?term=Liu+H&cauthor_id=37371833), 2023 | Necroptosis-Related Prognostic Model for Pancreatic Carcinoma Reveals Its Invasion and Metastasis Potential through Hybrid EMT and Immune Escape | Not related to the review objective and excluded by title and abstract |
| 34 | [Seval Müzeyyen Ecin](https://pubmed.ncbi.nlm.nih.gov/?term=Ecin+SM&cauthor_id=38065882), 2023 | Evaluation of the clinical and prognostic importance of infection parameters in thyroid cancers: A cross-sectional study | Not related to the review objective and excluded by title and abstract |
| 35 | [Xeni Provatopoulou](https://pubmed.ncbi.nlm.nih.gov/?term=Provatopoulou+X&cauthor_id=24794392), 2014 | Interleukins as markers of inflammation in malignant and benign thyroid disease | Not related to the review objective and excluded by title and abstract |
| 36 | [Ying Sun](https://pubmed.ncbi.nlm.nih.gov/?term=Sun+Y&cauthor_id=36510497), 2022 | To Explore the Inhibitory Mechanism of Quercetin in Thyroid Papillary Carcinoma through Network Pharmacology and Experiments | Not related to the review objective and excluded by title and abstract |
| 37 | [Muhammad Zubair Khan](https://pubmed.ncbi.nlm.nih.gov/?term=Zubair+Khan+M&cauthor_id=34621418), 2021 | Association of atrial fibrillation and various cancer subtypes | Not related to the review objective and excluded by title and abstract |
| 38 | [Nikita Pozdeyev](https://pubmed.ncbi.nlm.nih.gov/?term=Pozdeyev+N&cauthor_id=32242507) | Comprehensive Immune Profiling of Medullary Thyroid Cancer | Not related to the review objective and excluded by title and abstract |
| 39 | [Rilan Bai](https://pubmed.ncbi.nlm.nih.gov/?term=Bai+R&cauthor_id=34335763) , 2021 | Correlation of Peripheral Blood Parameters and Immune-Related Adverse Events with the Efficacy of Immune Checkpoint Inhibitors | Not related to the review objective and excluded by title and abstract |
| 40 | [Yifei Ma](https://pubmed.ncbi.nlm.nih.gov/?term=Ma+Y&cauthor_id=37090700) | Thyroid function and associated mood changes after COVID-19 vaccines in patients with Hashimoto thyroiditis | Not related to the review objective and excluded by title and abstract |
| 41 | [Valentina Bova](https://pubmed.ncbi.nlm.nih.gov/?term=Bova+V&cauthor_id=39200219) | The Protective Role of Troxerutin (Trox) in Counteracting Anaplastic Thyroid Carcinoma (ATC) Progression | Not related to the review objective and excluded by title and abstract |
| 42 | [Lingqian Zhao](https://pubmed.ncbi.nlm.nih.gov/?term=Zhao+L&cauthor_id=38189045) | Preoperative risk stratification for patients with ≤ 1 cm papillary thyroid carcinomas based on preoperative blood inflammatory markers: construction of a dynamic predictive model | Duplicate removed |
| 43 | [Roberta Modica](https://pubmed.ncbi.nlm.nih.gov/?term=Modica+R&cauthor_id=37373942), 2023 | Evaluation of Neutrophil-to-Lymphocyte Ratio (NLR), Platelet-to-Lymphocyte Ratio (PLR) and Systemic Immune-Inflammation Index (SII) as Potential Biomarkers in Patients with Sporadic Medullary Thyroid Cancer (MTC) | Not report outcome variables |
| 44 | [D G P N Villagelin](https://pubmed.ncbi.nlm.nih.gov/?term=Villagelin+DG&cauthor_id=21765238), 2011 | Is diffuse and peritumoral lymphocyte infiltration in papillary thyroid cancer a marker of good prognosis? | Not related to the review objective and excluded by title and abstract |
| 45 | [Tarek M Okda](https://pubmed.ncbi.nlm.nih.gov/?term=Okda+TM&cauthor_id=35203561), 2022 | A Novel Role of Galectin-3 and Thyroglobulin in Prognosis and Differentiation of Different Stages of Thyroid Cancer and Elucidation of the Potential Contribution of Bcl-2, IL-8 and TNF-α | Not related to the review objective and excluded by title and abstract |
| 46 | [Sumeet Suresh Malapure](https://pubmed.ncbi.nlm.nih.gov/?term=Malapure+SS&cauthor_id=38390543), 2023 | Association of Neutrophil-to-Lymphocyte Ratio and Lymphocyte-to-Monocyte Ratio with Clinicopathological Features and Short-Term Outcome in Well-Differentiated Thyroid Cancer | Not related to the review objective and excluded by title and abstract |
| 47 | [Bi-Cheng Wang](https://pubmed.ncbi.nlm.nih.gov/?term=Wang+BC&cauthor_id=30174788), 2018 | UHRF1 suppression promotes cell differentiation and reduces inflammatory reaction in anaplastic thyroid cancer | Not related to the review objective and excluded by title and abstract |
| 48 | [Yoon Kyoung So](https://pubmed.ncbi.nlm.nih.gov/?term=So+YK&cauthor_id=37509202), 2023 | Detection of Cancer Recurrence Using Systemic Inflammatory Markers and Machine Learning after Concurrent Chemoradiotherapy for Head and Neck Cancers | Not related to the review objective and excluded by title and abstract |
| 49 | [Deba Song](https://pubmed.ncbi.nlm.nih.gov/?term=Song+D&cauthor_id=35702097) | Expression and prognostic value of ratios of platelet lymphocyte, neutrophil lymphocyte and lymphocyte monocyte in breast cancer patients | Not related to the review objective and excluded by title and abstract |
| 50 | [Cínthia Minatel Riguetto](https://pubmed.ncbi.nlm.nih.gov/?term=Riguetto+CM&cauthor_id=34406270) , 2021 | Usefulness of pre-thyroidectomy neutrophil-lymphocyte, platelet-lymphocyte, and monocyte-lymphocyte ratios for discriminating lymph node and distant metastases in differentiated thyroid cancer | Not related to the review objective and excluded by title and abstract |
| 51 | [Deniz Tazeoglu](https://pubmed.ncbi.nlm.nih.gov/?term=Tazeoglu+D&cauthor_id=37324293), 2023 | Is it Possible to Diagnose "Non-Invasive Follicular Thyroid Neoplasm with Papillary-Like Nuclear Features" Preoperatively? | Not related to the review objective and excluded by title and abstract |
| 52 | [Cinthia Carolina Stempin](https://pubmed.ncbi.nlm.nih.gov/?term=Stempin+CC&cauthor_id=34638305), 2021 | Secreted Factors by Anaplastic Thyroid Cancer Cells Induce Tumor-Promoting M2-like Macrophage Polarization through a TIM3-Dependent Mechanism | Not related to the review objective and excluded by title and abstract |
| 53 | [Gulali Aktas](https://pubmed.ncbi.nlm.nih.gov/?term=Aktas+G&cauthor_id=29017671), 2017 | Could Red Cell Distribution Width be a Marker of Thyroid Cancer? | Not related to the review objective and excluded by title and abstract |
| 54 | [Adina Elena Stanciu](https://pubmed.ncbi.nlm.nih.gov/?term=Stanciu+AE&cauthor_id=35454805), 2022 | Analysis of the Correlation between the Radioactive Iodine Activity and Neutrophil-to-Lymphocyte Ratio in Patients with Differentiated Thyroid Cancer | Not related to the review objective and excluded by title and abstract |
| 55 | [Adina Elena Stanciu](https://pubmed.ncbi.nlm.nih.gov/?term=Stanciu+AE&cauthor_id=37568611) , 2023 | Portrait of the Inflammatory Response to Radioiodine Therapy in Female Patients with Differentiated Thyroid Cancer with/without Type 2 Diabetes Mellitus | Not related to the review objective and excluded by title and abstract |
| 56 | [Aya Sawa](https://pubmed.ncbi.nlm.nih.gov/?term=Sawa+A&cauthor_id=36099888) , 2022 | Absolute Lymphocyte Count as an Independent Prognostic Factor in Metastatic Breast Cancer: A Retrospective Study | Not related to the review objective and excluded by title and abstract |
| 57 | [Yeşim Ceylan](https://pubmed.ncbi.nlm.nih.gov/?term=Ceylan+Y&cauthor_id=30942057) , 2019 | The Correlation of Clinicopathological Findings and Neutrophil-to-Lymphocyte and Platelet-to-Lymphocyte Ratios in Papillary Thyroid Carcinoma | Not report outcome variables |
| 58 | [Kensey Bergdorf](https://pubmed.ncbi.nlm.nih.gov/?term=Bergdorf+K&cauthor_id=30965283), 2019 | Papillary thyroid carcinoma behavior: clues in the tumor microenvironment | Not related to the review objective and excluded by title and abstract |
| 59 | [Takashi Kijima](https://pubmed.ncbi.nlm.nih.gov/?term=Kijima+T&cauthor_id=27889946), 2017 | Combined fibrinogen and neutrophil-lymphocyte ratio as a prognostic marker of advanced esophageal squamous cell carcinoma | Not related to the review objective and excluded by title and abstract |
| 60 | [Qi Qi](https://pubmed.ncbi.nlm.nih.gov/?term=Qi+Q&cauthor_id=36251517), 2023 | Per- and polyfluoroalkyl substances activate UPR pathway, induce steatosis and fibrosis in liver cells | Duplicate removed |
| 61 | [Hakan Balbaloglu](https://pubmed.ncbi.nlm.nih.gov/?term=Balbaloglu+H&cauthor_id=38023432), 2023 | Predicting coexisting thyroid cancer with primary hyperparathyroidism in an endemic region of multinodular goiter: evaluating the effectiveness of preoperative inflammatory markers | Not related to the review objective and excluded by title and abstract |
| 62 | [Matthew H Taylor](https://pubmed.ncbi.nlm.nih.gov/?term=Taylor+MH&cauthor_id=33637020) , 2021 | Correlation of Performance Status and Neutrophil-Lymphocyte Ratio with Efficacy in Radioiodine-Refractory Differentiated Thyroid Cancer Treated with Lenvatinib | Not related to the review objective and excluded by title and abstract |
| 63 | [Danni Zheng](https://pubmed.ncbi.nlm.nih.gov/?term=Zheng+D&cauthor_id=36573167), 2022 | Fibrinogen-to-Neutrophil Ratio as a New Predictor of Central Lymph Node Metastasis in Patients with Papillary Thyroid Cancer and Type 2 Diabetes Mellitus | Not related to the review objective and excluded by title and abstract |
| 64 | [Chunying Peng](https://pubmed.ncbi.nlm.nih.gov/?term=Peng+C&cauthor_id=36839733), 2023 | Influence of Lenvatinib on the Functional Reprogramming of Peripheral Myeloid Cells in the Context of Non-Medullary Thyroid Carcinoma | Not related to the review objective and excluded by title and abstract |
| 65 | [Yanyi Huang](https://pubmed.ncbi.nlm.nih.gov/?term=Huang+Y&cauthor_id=35833862), 2022 | Inflammation Markers Have Important Value in Predicting Relapse in Patients with papillary thyroid carcinoma: A Long-Term Follow-Up Retrospective Study | Not related to the review objective and excluded by title and abstract |
| 66 | [Xing Zhen Liu](https://pubmed.ncbi.nlm.nih.gov/?term=Liu+XZ&cauthor_id=32164741) , 2020 | Monocyte-to-high-density lipoprotein cholesterol ratio is associated with the presence and size of thyroid nodule irrespective of the gender | Not related to the review objective and excluded by title and abstract |
| 67 | [Takaaki Arigami](https://pubmed.ncbi.nlm.nih.gov/?term=Arigami+T&cauthor_id=26893776), 2016 | Combined fibrinogen concentration and neutrophil-lymphocyte ratio as a prognostic marker of gastric cancer | Not related to the review objective and excluded by title and abstract |
| 68 | [Zhong-Wei Tang](https://pubmed.ncbi.nlm.nih.gov/?term=Tang+ZW&cauthor_id=37033338), 2023 | Development and validation of the nomogram based on ultrasound, thyroid stimulating hormone, and inflammatory marker in papillary thyroid carcinoma: a case-control study | Not related to the review objective and excluded by title and abstract |
| 69 | [Lucas Leite Cunha](https://pubmed.ncbi.nlm.nih.gov/?term=Cunha+LL&cauthor_id=27858102), 2017 | Interleukin 10 expression is related to aggressiveness and poor prognosis of patients with thyroid cancer | Not related to the review objective and excluded by title and abstract |
| 70 | [Tetsushi Hirahara](https://pubmed.ncbi.nlm.nih.gov/?term=Hirahara+T&cauthor_id=31286873) , 2019 | Combined neutrophil-lymphocyte ratio and platelet-lymphocyte ratio predicts chemotherapy response and prognosis in patients with advanced gastric cancer | Not related to the review objective and excluded by title and abstract |
| 71 | [Saliha Yildiz](https://pubmed.ncbi.nlm.nih.gov/?term=Yildiz+S&cauthor_id=31740871) , 2019 | A comparison of haemogram parameters of patients with thyroid papillary cancer and nodular goiter in Van, Turkey | Not related to the review objective and excluded by title and abstract |
| 72 | [Tommaso Piticchio](https://pubmed.ncbi.nlm.nih.gov/?term=Piticchio+T&cauthor_id=38275410), 2024 | Inflammatory Profile Assessment in a Highly Selected Athyreotic Population Undergoing Controlled and Standardized Hypothyroidism | Not related to the review objective and excluded by title and abstract |
| 73 | [Fatma El Zahraa A Mohamed](https://pubmed.ncbi.nlm.nih.gov/?term=Mohamed+FEZA&cauthor_id=33846395), 2021 | Thyroid hormone receptor α1 acts as a new squamous cell lung cancer diagnostic marker and poor prognosis predictor | Not related to the review objective and excluded by title and abstract |
| 74 | [A V Isaeva](https://pubmed.ncbi.nlm.nih.gov/?term=Isaeva+AV&cauthor_id=26710532), 2015 | [β-Catenin: Structure, Function and Role in Malignant Transformation of Epithelial Cells] | Not related to the review objective and excluded by title and abstract |
| 75 | [Ke Jiang](https://pubmed.ncbi.nlm.nih.gov/?term=Jiang+K&cauthor_id=27749581), 2016 | Association of the preoperative neutrophil-to-lymphocyte and platelet-to-lymphocyte ratios with lymph node metastasis and recurrence in patients with medullary thyroid carcinoma | Not related to the review objective and excluded by title and abstract |
| 76 | [Antonio Macciò](https://pubmed.ncbi.nlm.nih.gov/?term=Macci%C3%B2+A&cauthor_id=25239265) , 2015 | The role of inflammation, iron, and nutritional status in cancer-related anemia: results of a large, prospective, observational study | Not related to the review objective and excluded by title and abstract |
| 77 | [Shogo Nakamoto](https://pubmed.ncbi.nlm.nih.gov/?term=Nakamoto+S&cauthor_id=35403159), 2021 | The Systemic Immune Markers at Diagnosis Can Predict the Survival Benefit in Advanced Breast Cancer | Duplicate removed |
| 78 | [Behnaz Karkheiran](https://pubmed.ncbi.nlm.nih.gov/?term=Karkheiran+B&cauthor_id=36936544), 2023 | Evaluation of PD-L1 antigen expression using immunohistochemistry technique in medullary thyroid carcinoma samples | Not related to the review objective and excluded by title and abstract |
| 79 | [Yukyung Hong](https://pubmed.ncbi.nlm.nih.gov/?term=Hong+Y&cauthor_id=34208730) , 2021 | Quercetin Induces Anticancer Activity by Upregulating Pro-NAG-1/GDF15 in Differentiated Thyroid Cancer Cells | Not related to the review objective and excluded by title and abstract |
| 80 | [Hyo Jung An](https://pubmed.ncbi.nlm.nih.gov/?term=An+HJ&cauthor_id=28994272), 2018 | Programmed Death-Ligand 1 Expression and Its Correlation with Lymph Node Metastasis in Papillary Thyroid Carcinoma | Not related to the review objective and excluded by title and abstract |
| 81 | [Josephine H Li](https://pubmed.ncbi.nlm.nih.gov/?term=Li+JH&cauthor_id=33997632), 2021 | Painful Subacute Thyroiditis is Commonly Misdiagnosed as Suspicious Thyroid Nodular Disease | Not related to the review objective and excluded by title and abstract |
| 82 | [Yanhua Yang](https://pubmed.ncbi.nlm.nih.gov/?term=Yang+Y&cauthor_id=32151175), 2020 | MiR-324-5p/PTPRD/CEBPD axis promotes papillary thyroid carcinoma progression via microenvironment alteration | Not related to the review objective and excluded by title and abstract |
| 83 | [Ismael Reyes](https://pubmed.ncbi.nlm.nih.gov/?term=Reyes+I&cauthor_id=30614796), 2019 | Gene expression profiling identifies potential molecular markers of papillary thyroid carcinoma | Not related to the review objective and excluded by title and abstract |
| 84 | [Alessia Dolci](https://pubmed.ncbi.nlm.nih.gov/?term=Dolci+A&cauthor_id=31057489), 2019 | Post-surgical Thyroid Bed Pyoderma Gangrenosum Mimicking Recurrent Papillary Thyroid Carcinoma | Not related to the review objective and excluded by title and abstract |
| 85 | [Shogo Nakamoto](https://pubmed.ncbi.nlm.nih.gov/?term=Nakamoto+S&cauthor_id=37904008), 2023 | Systemic immunity markers are associated with clinical outcomes of atezolizumab treatment in patients with triple-negative advanced breast cancer: a retrospective multicenter observational study | Not related to the review objective and excluded by title and abstract |
| 86 | [Hanna J Lee](https://pubmed.ncbi.nlm.nih.gov/?term=Lee+HJ&cauthor_id=37168978) , 2023 | Permanent hypothyroidism following immune checkpoint inhibitors induced thyroiditis may be associated with improved survival: results of an exploratory study | Not related to the review objective and excluded by title and abstract |
| 87 | [Jian-Guo Zhou](https://pubmed.ncbi.nlm.nih.gov/?term=Zhou+JG&cauthor_id=35844547), 2022 | Elucidation of the Application of Blood Test Biomarkers to Predict Immune-Related Adverse Events in Atezolizumab-Treated NSCLC Patients Using Machine Learning Methods | Not related to the review objective and excluded by title and abstract |
| 88 | [Shogo Nakamoto](https://pubmed.ncbi.nlm.nih.gov/?term=Nakamoto+S&cauthor_id=35403159), 2021 | The Systemic Immune Markers at Diagnosis Can Predict the Survival Benefit in Advanced Breast Cancer | Not related to the review objective and excluded by title and abstract |
| 89 | [Tiziana Di Marco](https://pubmed.ncbi.nlm.nih.gov/?term=Di+Marco+T&cauthor_id=32061953), 2020 | COPZ1 depletion in thyroid tumor cells triggers type I IFN response and immunogenic cell death | Not related to the review objective and excluded by title and abstract |
| 90 | [Ashish V Chintakuntlawar](https://pubmed.ncbi.nlm.nih.gov/?term=Chintakuntlawar+AV&cauthor_id=28324060) , 2017 | Expression of PD-1 and PD-L1 in Anaplastic Thyroid Cancer Patients Treated With Multimodal Therapy: Results From a Retrospective Study | Not related to the review objective and excluded by title and abstract |
| 91 | [Matthieu-John Ouvrier](https://pubmed.ncbi.nlm.nih.gov/?term=Ouvrier+MJ&cauthor_id=24153039) , 2013 | [State of the art in nuclear imaging for the diagnosis of bone metastases] | Not related to the review objective and excluded by title and abstract |
| 92 | [J McAllister](https://pubmed.ncbi.nlm.nih.gov/?term=McAllister+J&cauthor_id=34448666), 2022 | Spontaneous thyroid abscesses without underlying malignancy: a differential diagnosis for recent onset or rapidly growing thyroid masses | Not related to the review objective and excluded by title and abstract |
| 93 | [Michael Ivanovich Sheremet](https://pubmed.ncbi.nlm.nih.gov/?term=Sheremet+MI&cauthor_id=29250672), 2017 | Association of the blood serum cytokines' rate and lymphocytes' apoptosis with polymorphic variants of the BCL-2 (rs17759659), CTLA-4 (rs231775) and APO-1÷FAS (rs2234767) genes in patients with nodular goiters in autoimmune thyroiditis and thyroid adenoma | Not related to the review objective and excluded by title and abstract |
| 94 | [Ju-Yeon Kim](https://pubmed.ncbi.nlm.nih.gov/?term=Kim+JY&cauthor_id=24272600), 2014 | Prognostic importance of baseline neutrophil to lymphocyte ratio in patients with advanced papillary thyroid carcinomas | Not related to the review objective and excluded by title and abstract |
| 95 | [Kwan Ho Lee](https://pubmed.ncbi.nlm.nih.gov/?term=Lee+KH&cauthor_id=30746354) , 2019 | Different prognostic values of individual hematologic parameters in papillary thyroid cancer due to age-related changes in immunity | Not related to the review objective and excluded by title and abstract |
| 96 | [K E Zhang](https://pubmed.ncbi.nlm.nih.gov/?term=Zhang+KE&cauthor_id=26998068), 2016 | Intercellular adhesion molecule 1 is a sensitive and diagnostically useful immunohistochemical marker of papillary thyroid cancer (PTC) and of PTC-like nuclear alterations in Hashimoto's thyroiditis | Not related to the review objective and excluded by title and abstract |
| 97 | [Adeena Khan](https://pubmed.ncbi.nlm.nih.gov/?term=Khan+A&cauthor_id=34007385) | Thyroid "claw sign" a useful diagnostic marker in the outsized lesions of isthmus: A large colloid cyst | Not related to the review objective and excluded by title and abstract |
| 98 | [Mark C Royer](https://pubmed.ncbi.nlm.nih.gov/?term=Royer+MC&cauthor_id=20231640) , 2010 | Genetic alterations in papillary thyroid carcinoma and hashimoto thyroiditis: An analysis of hOGG1 loss of heterozygosity | Not related to the review objective and excluded by title and abstract |
| 99 | [Takaaki Arigami](https://pubmed.ncbi.nlm.nih.gov/?term=Arigami+T&cauthor_id=26496280), 2015 | Analysis of the Fibrinogen and Neutrophil-Lymphocyte Ratio in Esophageal Squamous Cell Carcinoma: A Promising Blood Marker of Tumor Progression and Prognosis | Not related to the review objective and excluded by title and abstract |
| 100 | [Mengliu Zhu](https://pubmed.ncbi.nlm.nih.gov/?term=Zhu+M&cauthor_id=35295846) , 2022 | The Systemic Inflammation Response Index as an Independent Predictor of Survival in Breast Cancer Patients: A Retrospective Study | Not related to the review objective and excluded by title and abstract |
| 101 | [Sabahattin Destek](https://pubmed.ncbi.nlm.nih.gov/?term=Destek+S&cauthor_id=35990292), 2022 | Clinical Significance of Erythrocyte Sedimentation Rate, Leukocyte, Fibrinogen, C-Reactive Protein, and Pentraxin 3 Values in Thyroid Nodules | Not related to the review objective and excluded by title and abstract |
| 102 | [Takaaki Arigami](https://pubmed.ncbi.nlm.nih.gov/?term=Arigami+T&cauthor_id=26981856), 2016 | A Novel Scoring System Based on Fibrinogen and the Neutrophil-Lymphocyte Ratio as a Predictor of Chemotherapy Response and Prognosis in Patients with Advanced Gastric Cancer | Not related to the review objective and excluded by title and abstract |
| 103 | [Angelica Perna](https://pubmed.ncbi.nlm.nih.gov/?term=Perna+A&cauthor_id=29448931), 2018 | Effects of different extracts of curcumin on TPC1 papillary thyroid cancer cell line | Not related to the review objective and excluded by title and abstract |
| 104 | [Zhihong Deng](https://pubmed.ncbi.nlm.nih.gov/?term=Deng+Z&cauthor_id=34113406), 2021 | Combination Model of Thyrotrophin Receptor Antibody and Volumetric Orbital Apex Crowding Index as an Indicator of Dysthyroid Optic Neuropathy | Not related to the review objective and excluded by title and abstract |
| 105 | [Mara Mazzoni](https://pubmed.ncbi.nlm.nih.gov/?term=Mazzoni+M&cauthor_id=31113465), 2019 | Senescent thyrocytes and thyroid tumor cells induce M2-like macrophage polarization of human monocytes via a PGE2-dependent mechanism | Not related to the review objective and excluded by title and abstract |
| 106 | [Yoonjoo Kim](https://pubmed.ncbi.nlm.nih.gov/?term=Kim+Y&cauthor_id=36532699), 2022 | The Tri-iodothyronine (T3) Level Is a Prognostic Factor for Patients With Advanced NSCLC: Receiving Immune Checkpoint Inhibitors and Is Associated With Liver Metastasis | Not related to the review objective and excluded by title and abstract |
| 107 | [Laura Gianotti](https://pubmed.ncbi.nlm.nih.gov/?term=Gianotti+L&cauthor_id=33997278), 2021 | Persistence of Elevated Procalcitonin in a Patient with Coronavirus Disease 2019 Uncovered a Diagnosis of Medullary Thyroid Carcinoma | Not related to the review objective and excluded by title and abstract |
| 108 | [Tao Zhou](https://pubmed.ncbi.nlm.nih.gov/?term=Zhou+T&cauthor_id=30719156), 2019 | Pretreatment albumin globulin ratio has a superior prognostic value in laryngeal squamous cell carcinoma patients: a comparison study | Not related to the review objective and excluded by title and abstract |
| 109 | [Z Tsagareli](https://pubmed.ncbi.nlm.nih.gov/?term=Tsagareli+Z&cauthor_id=28009314), 2016 | HURTLE CELLS IMMUNOHISTOCHEMICAL ACTIVITIES IN HASHIMOTO THYROIDITIS PARENCHYMA | Not related to the review objective and excluded by title and abstract |
| 110 | [Hao Sun](https://pubmed.ncbi.nlm.nih.gov/?term=Sun+H&cauthor_id=36438745), 2022 | Prognostic nutritional index for predicting the clinical outcomes of patients with gastric cancer who received immune checkpoint inhibitors | Not related to the review objective and excluded by title and abstract |
| 111 | [Nina Mikirova](https://pubmed.ncbi.nlm.nih.gov/?term=Mikirova+N&cauthor_id=22963460), 2012 | Effect of high-dose intravenous vitamin C on inflammation in cancer patients | Not related to the review objective and excluded by title and abstract |
| 112 | [Lavanya Raman](https://pubmed.ncbi.nlm.nih.gov/?term=Raman+L&cauthor_id=24577178), 2014 | Primary tuberculosis of the thyroid gland: an unexpected cause of thyrotoxicosis | Not related to the review objective and excluded by title and abstract |
| 113 | [R S Redman](https://pubmed.ncbi.nlm.nih.gov/?term=Redman+RS&cauthor_id=31423894) , 2019 | Salivary and serum biomarkers of inflammation in a man with metastatic medullary thyroid carcinoma and hyperreactive gingiva: a fourteen year odyssey | Not related to the review objective and excluded by title and abstract |
| 114 | [Salvatore Sciacchitano](https://pubmed.ncbi.nlm.nih.gov/?term=Sciacchitano+S&cauthor_id=33794925), 2021 | Gene signature and immune cell profiling by high-dimensional, single-cell analysis in COVID-19 patients, presenting Low T3 syndrome and coexistent hematological malignancies | Not related to the review objective and excluded by title and abstract |
| 115 | [Guglielmo Ardito](https://pubmed.ncbi.nlm.nih.gov/?term=Ardito+G&cauthor_id=20565793), 2010 | Immunohistochemical evaluation of inflammatory and proliferative markers in adjacent normal thyroid tissue in patients undergoing total thyroidectomy: results of a preliminary study | Not related to the review objective and excluded by title and abstract |
| 116 | [Tal Sigawi](https://pubmed.ncbi.nlm.nih.gov/?term=Sigawi+T&cauthor_id=39139285), 2024 | Improving the response to lenvatinib in partial responders using a Constrained-Disorder-Principle-based second-generation artificial intelligence-therapeutic regimen: a proof-of-concept open-labeled clinical trial | Not related to the review objective and excluded by title and abstract |
| 117 | [Neal M Dixit](https://pubmed.ncbi.nlm.nih.gov/?term=Dixit+NM&cauthor_id=32984743), 2020 | Sudden Cardiac Arrest in a Patient with Myxedema Coma and COVID-19 | Not related to the review objective and excluded by title and abstract |
| 118 | [Kinga Krawczyk-Rusiecka](https://pubmed.ncbi.nlm.nih.gov/?term=Krawczyk-Rusiecka+K&cauthor_id=22419920), 2010 | Cyclooxygenase-2 expression and its association with thyroid lesions | Not related to the review objective and excluded by title and abstract |
| 119 | [Guo-Wen Lu](https://pubmed.ncbi.nlm.nih.gov/?term=Lu+GW&cauthor_id=32529881), 2020 | Usefulness of postoperative serum translocator protein as a predictive marker for delirium after breast cancer surgery in elderly women | Not related to the review objective and excluded by title and abstract |
| 120 | [Guillaume Ulmann](https://pubmed.ncbi.nlm.nih.gov/?term=Ulmann+G&cauthor_id=31865308), 2019 | Lean Body Mass and Endocrine Status But Not Age Are Determinants of Resting Energy Expenditure in Patients with Non-Small Cell Lung Cancer | Not related to the review objective and excluded by title and abstract |
| 121 | [Noor Mohammed Al-Timimi](https://pubmed.ncbi.nlm.nih.gov/?term=Al-Timimi+NM&cauthor_id=39868448), 2024 | KI-67 as a predictive indicator of papillary thyroid cancer in Iraqi patients | Not related to the review objective and excluded by title and abstract |
| 122 | [Mustafa C Şenoymak](https://pubmed.ncbi.nlm.nih.gov/?term=%C5%9Eenoymak+MC&cauthor_id=38862101), 2024 | Assessment of inflammatory parameters as predictive markers for malignancy in thyroid nodules: a study on the correlation with Bethesda classification | Not related to the review objective and excluded by title and abstract |
| 123 | [Suleyman Baldane](https://pubmed.ncbi.nlm.nih.gov/?term=Baldane+S&cauthor_id=25854344), 2015 | Mean platelet volume could be a possible biomarker for papillary thyroid carcinomas | Not related to the review objective and excluded by title and abstract |
| 124 | [Ju-Yeon Kim](https://pubmed.ncbi.nlm.nih.gov/?term=Kim+JY&cauthor_id=24272600), 2014 | Prognostic importance of baseline neutrophil to lymphocyte ratio in patients with advanced papillary thyroid carcinomas | Duplicate removed |
| 125 | [Kwan Ho Lee](https://pubmed.ncbi.nlm.nih.gov/?term=Lee+KH&cauthor_id=30746354) , 2019 | Different prognostic values of individual hematologic parameters in papillary thyroid cancer due to age-related changes in immunity | Duplicate removed |
| 126 | [K E Zhang](https://pubmed.ncbi.nlm.nih.gov/?term=Zhang+KE&cauthor_id=26998068), 2016 | Intercellular adhesion molecule 1 is a sensitive and diagnostically useful immunohistochemical marker of papillary thyroid cancer (PTC) and of PTC-like nuclear alterations in Hashimoto's thyroiditis | Duplicate removed |
| 127 | [Adeena Khan](https://pubmed.ncbi.nlm.nih.gov/?term=Khan+A&cauthor_id=34007385), 2021 | Thyroid "claw sign" a useful diagnostic marker in the outsized lesions of isthmus: A large colloid cyst | Duplicate removed |
| 128 | [Mark C Royer](https://pubmed.ncbi.nlm.nih.gov/?term=Royer+MC&cauthor_id=20231640) , 2010 | Genetic alterations in papillary thyroid carcinoma and hashimoto thyroiditis: An analysis of hOGG1 loss of heterozygosity | Duplicate removed |
| 129 | [Takaaki Arigami](https://pubmed.ncbi.nlm.nih.gov/?term=Arigami+T&cauthor_id=26496280), 2015 | Analysis of the Fibrinogen and Neutrophil-Lymphocyte Ratio in Esophageal Squamous Cell Carcinoma: A Promising Blood Marker of Tumor Progression and Prognosis | Duplicate removed |
| 130 | [Mengliu Zhu](https://pubmed.ncbi.nlm.nih.gov/?term=Zhu+M&cauthor_id=35295846) , 2022 | The Systemic Inflammation Response Index as an Independent Predictor of Survival in Breast Cancer Patients: A Retrospective Study | Duplicate removed |
| 131 | [Sabahattin Destek](https://pubmed.ncbi.nlm.nih.gov/?term=Destek+S&cauthor_id=35990292), 2022 | Clinical Significance of Erythrocyte Sedimentation Rate, Leukocyte, Fibrinogen, C-Reactive Protein, and Pentraxin 3 Values in Thyroid Nodules | Duplicate removed |
| 132 | [Takaaki Arigami](https://pubmed.ncbi.nlm.nih.gov/?term=Arigami+T&cauthor_id=26981856), 2016 | A Novel Scoring System Based on Fibrinogen and the Neutrophil-Lymphocyte Ratio as a Predictor of Chemotherapy Response and Prognosis in Patients with Advanced Gastric Cancer | Duplicate removed |
| 133 | [Ju-Yeon Kim](https://pubmed.ncbi.nlm.nih.gov/?term=Kim+JY&cauthor_id=24272600), 2014 | Prognostic importance of baseline neutrophil to lymphocyte ratio in patients with advanced papillary thyroid carcinomas | Duplicate removed |
| 134 | [Kwan Ho Lee](https://pubmed.ncbi.nlm.nih.gov/?term=Lee+KH&cauthor_id=30746354) , 2019 | Different prognostic values of individual hematologic parameters in papillary thyroid cancer due to age-related changes in immunity | Duplicate removed |
| 135 | [K E Zhang](https://pubmed.ncbi.nlm.nih.gov/?term=Zhang+KE&cauthor_id=26998068), 2016 | Intercellular adhesion molecule 1 is a sensitive and diagnostically useful immunohistochemical marker of papillary thyroid cancer (PTC) and of PTC-like nuclear alterations in Hashimoto's thyroiditis | Duplicate removed |
| 136 | [Adeena Khan](https://pubmed.ncbi.nlm.nih.gov/?term=Khan+A&cauthor_id=34007385) | Thyroid "claw sign" a useful diagnostic marker in the outsized lesions of isthmus: A large colloid cyst | Duplicate removed |
| 137 | [Mark C Royer](https://pubmed.ncbi.nlm.nih.gov/?term=Royer+MC&cauthor_id=20231640) , 2010 | Genetic alterations in papillary thyroid carcinoma and hashimoto thyroiditis: An analysis of hOGG1 loss of heterozygosity | Duplicate removed |
| 138 | [Takaaki Arigami](https://pubmed.ncbi.nlm.nih.gov/?term=Arigami+T&cauthor_id=26496280), 2015 | Analysis of the Fibrinogen and Neutrophil-Lymphocyte Ratio in Esophageal Squamous Cell Carcinoma: A Promising Blood Marker of Tumor Progression and Prognosis | Duplicate removed |
| 139 | [Mengliu Zhu](https://pubmed.ncbi.nlm.nih.gov/?term=Zhu+M&cauthor_id=35295846) , 2022 | The Systemic Inflammation Response Index as an Independent Predictor of Survival in Breast Cancer Patients: A Retrospective Study | Duplicate removed |
| 140 | [Sabahattin Destek](https://pubmed.ncbi.nlm.nih.gov/?term=Destek+S&cauthor_id=35990292), 2022 | Clinical Significance of Erythrocyte Sedimentation Rate, Leukocyte, Fibrinogen, C-Reactive Protein, and Pentraxin 3 Values in Thyroid Nodules | Duplicate removed |
| 141 | [Marra Jai Aghajani](https://pubmed.ncbi.nlm.nih.gov/?term=Aghajani+MJ&cauthor_id=33112841) , 2020 | Epithelial-to-mesenchymal transition and its association with PD-L1 and CD8 in thyroid cancer | Duplicate removed |
| 142 | [Ashank Bains](https://pubmed.ncbi.nlm.nih.gov/?term=Bains+A&cauthor_id=34298730), 2021 | The Role of Vitamin D as a Prognostic Marker in Papillary Thyroid Cancer | Duplicate removed |
| 143 | [Hyunjae Lee](https://pubmed.ncbi.nlm.nih.gov/?term=Lee+H&cauthor_id=37909732), 2023 | Value of circulating neutrophil elastase for detecting recurrence of differentiated thyroid cancer | Duplicate removed |
| 144 | [Jiyun Park](https://pubmed.ncbi.nlm.nih.gov/?term=Park+J&cauthor_id=33921107), 2021 | Prognostic Value of the Neutrophil-to-Lymphocyte Ratio before and after Radiotherapy for Anaplastic Thyroid Carcinoma | Duplicate removed |
| 145 | [Ashank Bains](https://pubmed.ncbi.nlm.nih.gov/?term=Bains+A&cauthor_id=34298730), 2021 | The Role of Vitamin D as a Prognostic Marker in Papillary Thyroid Cancer | Duplicate removed |
| 146 | [Natália Medeiros Dias Lopes](https://pubmed.ncbi.nlm.nih.gov/?term=Lopes+NMD&cauthor_id=35902455) ,2022 | Role of papillary thyroid carcinoma patients with Hashimoto thyroiditis: evaluation of oxidative stress and inflammatory markers | Duplicate removed |
| 147 | [Chiara Offi](https://pubmed.ncbi.nlm.nih.gov/?term=Offi+C&cauthor_id=33746220), 2021 | Clinical significance of neutrophil-to-lymphocyte ratio, lymphocyte-to-monocyte ratio, platelet-to-lymphocyte ratio and prognostic nutritional index in low-risk differentiated thyroid carcinoma | Duplicate removed |
| 148 | [Qi Qi](https://pubmed.ncbi.nlm.nih.gov/?term=Qi+Q&cauthor_id=36251517), 2023 | Per- and polyfluoroalkyl substances activate UPR pathway, induce steatosis and fibrosis in liver cells | Duplicate removed |
| 149 | [Lingqian Zhao](https://pubmed.ncbi.nlm.nih.gov/?term=Zhao+L&cauthor_id=38189045), 2023 | Preoperative risk stratification for patients with ≤ 1 cm papillary thyroid carcinomas based on preoperative blood inflammatory markers: construction of a dynamic predictive model | Duplicate removed |
| 150 | [Selahattin Vural](https://pubmed.ncbi.nlm.nih.gov/?term=Vural+S&cauthor_id=37543770), 2023 | Systemic immune-inflammation index: A new marker in differentiation of different thyroid diseases | Duplicate removed |
| 151 | [Arunangshu Ghoshal](https://pubmed.ncbi.nlm.nih.gov/?term=Ghoshal+A&cauthor_id=29416653) , 2017 | Thyroid cancer risk in the Swedish AMORIS study: the role of inflammatory biomarkers in serum | Duplicate removed |
| 152 | [F De Paoli](https://pubmed.ncbi.nlm.nih.gov/?term=De+Paoli+F&cauthor_id=25941992), 2015 | The neuron-derived orphan receptor 1 (NOR1) is induced upon human alternative macrophage polarization and stimulates the expression of markers of the M2 phenotype | Duplicate removed |
| 153 | [Marra Jai Aghajani](https://pubmed.ncbi.nlm.nih.gov/?term=Aghajani+MJ&cauthor_id=33112841) , 2020 | Epithelial-to-mesenchymal transition and its association with PD-L1 and CD8 in thyroid cancer | Duplicate removed |
| 154 | [Dehui Qiao](https://pubmed.ncbi.nlm.nih.gov/?term=Qiao+D&cauthor_id=38568295) , 2024 | Nomogram to predict central lymph node metastasis in papillary thyroid carcinoma | Duplicate removed |
| 155 | [Hyunjae Lee](https://pubmed.ncbi.nlm.nih.gov/?term=Lee+H&cauthor_id=37909732), 2023 | Value of circulating neutrophil elastase for detecting recurrence of differentiated thyroid cancer | Duplicate removed |
| 156 | [Jiyun Park](https://pubmed.ncbi.nlm.nih.gov/?term=Park+J&cauthor_id=33921107), 2021 | Prognostic Value of the Neutrophil-to-Lymphocyte Ratio before and after Radiotherapy for Anaplastic Thyroid Carcinoma | Duplicate removed |
| 157 | [Ashank Bains](https://pubmed.ncbi.nlm.nih.gov/?term=Bains+A&cauthor_id=34298730), 2021 | The Role of Vitamin D as a Prognostic Marker in Papillary Thyroid Cancer | Duplicate removed |
| 158 | [Natália Medeiros Dias Lopes](https://pubmed.ncbi.nlm.nih.gov/?term=Lopes+NMD&cauthor_id=35902455) ,2022 | Role of papillary thyroid carcinoma patients with Hashimoto thyroiditis: evaluation of oxidative stress and inflammatory markers | Duplicate removed |
| 159 | [Chiara Offi](https://pubmed.ncbi.nlm.nih.gov/?term=Offi+C&cauthor_id=33746220), 2021 | Clinical significance of neutrophil-to-lymphocyte ratio, lymphocyte-to-monocyte ratio, platelet-to-lymphocyte ratio and prognostic nutritional index in low-risk differentiated thyroid carcinoma | Duplicate removed |
| 160 | Dimitrios K. Manatakis, 2018 | Diagnostic Accuracy of Preoperative Neutrophil-to-Lymphocyte and Platelet-to-Lymphocyte Ratios in Detecting Occult Papillary Thyroid Microcarcinomas in Benign Multinodular Goitres | Duplicate removed |
| 161 | Derya Kocer, 2015 | May the Neutrophil/Lymphocyte Ratio Be a Predictor in the Differentiation of Different Thyroid Disorders? | Duplicate removed |
| 162 | Hayri Bostan, 2022 | The predictive value of hematologic parameters in the risk of thyroid malignancy in cases with atypia/follicular lesion of undetermined significance | Duplicate removed |
| 163 | Haider Salim Mihson, 2022 | The Role of Neutrophil to Lymphocyte and Platelet to Lymphocyte Ratios in Diagnosing Thyroid Nodule | Duplicate removed |
| 164 | Chiara Off, 2021 | Evaluation of LMR, NLR and PLR as predictors of malignancy in indeterminate thyroid nodules | Duplicate removed |
| 165 | Mehmet Bug˘ra Bozan, 2020 | Delta Neutrophil Index and Neutrophil-to-Lymphocyte Ratio in the Differentiation of Thyroid Malignancy and Nodular Goiter | Duplicate removed |
| 166 | [Roberta Modica](https://pubmed.ncbi.nlm.nih.gov/?term=Modica+R&cauthor_id=37373942), 2023 | Evaluation of Neutrophil-to-Lymphocyte Ratio (NLR), Platelet-to-Lymphocyte Ratio (PLR) and Systemic Immune-Inflammation Index (SII) as Potential Biomarkers in Patients with Sporadic Medullary Thyroid Cancer (MTC) | Not report outcome variables |
| 167 | [Selcuk Yaylaci](https://pubmed.ncbi.nlm.nih.gov/?term=Yaylaci+S&cauthor_id=27221938), 2016 | Lack of Variation in Inflammatory Hematological Parameters between Benign Nodular Goiter and Papillary Thyroid Cancer | Not report outcome variables |
| 168 | [Faruk Kutluturk](https://pubmed.ncbi.nlm.nih.gov/?term=Kutluturk+F&cauthor_id=30727930), 2019 | Comparison of Mean Platelet Volume, Platelet Count, Neutrophil/ Lymphocyte Ratio and Platelet/Lymphocyte Ratio in the Euthyroid, Overt Hypothyroid and Subclinical Hyperthyroid Phases of Papillary Thyroid Carcinoma | Not report outcome variables |
| 169 | [Nikolaos Machairas](https://pubmed.ncbi.nlm.nih.gov/?term=Machairas+N&cauthor_id=28506088) , 2017 | Trends in white blood cell and platelet indices in a comparison of patients with papillary thyroid carcinoma and multinodular goiter do not permit differentiation between the conditions | Not report outcome variables |
