# Supplementary material for: Diagnostic accuracy of neutrophil-to-lymphocyte and platelet-to-lymphocyte ratios in differentiating thyroid tumors: A systematic review and meta-analysis
Source: PLoS One. 2025 May 5;20(5):e0322382. doi: 10.1371/journal.pone.0322382 (PMC12052148; doi:10.1371/journal.pone.0322382)
Supplement: S4 File — (DOCX) [file pone.0322382.s004.docx]

**Table4: Summary characteristics of articles included in the systematic review (N = 12)**

| **Author, year** | **Mean age** | **Gender** | | **Study design** | **Sample size** | | | **Sensitivity (%) NLR** | **Specificity (%) NLR** | **Sensitivity (%)PLR** | **Specificity (%)PLR** | **Data extracted by** | **Date of data extraction** |
| --- | --- | --- | --- | --- | --- | --- | --- | --- | --- | --- | --- | --- | --- |
|  |  | **Male** | **Female** |  | **Adenoma** | **Carcinoma** | **Total** |  |  |  |  |  |  |
| Mehmet Bug˘ra Bozan, 2020 | 50 | 49 | 194 | retrospective cohort | 184 | 50 | 234 | 62 | 49 | - | - | Teketelew BB. And Mekuanint A. | Aug. 16, 2024 |
| Muzaffer Serdar Deniz, 2023 | 51.5±13.5 | 92 | 367 | retrospective cohort | 438 | 21 | 459 | 62 | 75 | 71 | 71 | Teketelew BB. and Berta DM. | Aug16, 2024 |
| Yuanyuan Deng, 2022 | 48 | 103 | 412 | retrospective | 374 | 140 | 514 | - | - | 54 | 62 | Cherie N. and Angelo AA. | Sept. 23, 2024 |
| Dimitrios K. Manatakis, 2018 | 53.01 ± 14.5 | 94 | 303 | retrospective | 190 | 207 | 397 | 80 | 34 | 73 | 37 | Chane E. and Cherie N | Oct. 12, 2024 |
| Derya Kocer, 2015 | 50.41±12.45 | 89 | 143 | retrospective | 167 | 65 | 232 | 89 | 54 | - | - | Walle M. and Alemayehu TT. | Oct. 20, 2024 |
| Satriya Kelana, 2022 | - | 15 | 47 | retrospective cohort | 10 | 52 | 62 | 69 | 70 | - | - | Mulatie Z. and Berta DM. | Sept. 3, 2024 |
| Hakan Bölükbaş, 2020 | 48.01±12.23 | 105 | 508 | retrospective | 417 | 196 | 613 | 82 | 33 | - | - | Teketelew BB. and Chane E. | Oct. 19, 2024 |
| Burcin Meryem Atak Tel, 2021 | 43.85±9.5 | 104 | 339 | retrospective | 207 | 236 | 443 | - | - | 69 | 51 | Teketelew BB. And Chane E. | Apr 3, 2024 |
| Hayri Bostan, 2022 | 48.3±12.3 | 25 | 175 | retrospective | 78 | 122 | 200 | 49 | 81 | - | - | Cherie N. and Angelo AA | Apr 3, 2024 |
| Mustafa C Şenoymak, 2024 | 53 medians | 123 | 484 | prospective cohort | 573 | 34 | 607 | 82 | 83 | - | - | Walle M. and Alemayehu TT. | Mar 27, 2024 |
| Haider Salim Mihson, 2022 | 52.65±16.26 | 15 | 67 | cohort | 49 | 33 | 82 | 85 | 71 | 88 | 71 | Chane E. and Cherie N | Apr 4, 2024 |
| Chiara Off, 2021 | 50.69 ± 14.73 | 63 | 235 | retrospective | 239 | 59 | 298 | - | - | 69 | 48 | Mulatie Z. and Berta DM. | Mar 29, 2024 |
