# Supplementary material for: Diagnostic accuracy of neutrophil-to-lymphocyte and platelet-to-lymphocyte ratios in differentiating thyroid tumors: A systematic review and meta-analysis
Source: PLoS One. 2025 May 5;20(5):e0322382. doi: 10.1371/journal.pone.0322382 (PMC12052148; doi:10.1371/journal.pone.0322382)
Supplement: S5 File — (DOCX) [file pone.0322382.s005.docx]

**Table 5. List of included articles in the final systemic review and meta-analysis**

| **S/N** | **Author Year** | **Articles with full title** |
| --- | --- | --- |
| 1 | Mehmet Bug˘ra Bozan, 2020 | Delta Neutrophil Index and Neutrophil-to-Lymphocyte Ratio in the Differentiation of Thyroid Malignancy and Nodular Goiter |
| 2 | Muzaffer Serdar Deniz, 2023 | A novel proportional index to differentiate between demographically and clinically matched cases with papillary thyroid carcinoma or non-cancerous nodule: PLR-to-PDW rati |
| 3 | Yuanyuan Deng, 2022 | Peripheral Blood Inflammatory Markers Can Predict Benign and Malignant Thyroid Nodules |
| 4 | Dimitrios K. Manatakis, 2018 | Diagnostic Accuracy of Preoperative Neutrophil-to-Lymphocyte and Platelet-to-Lymphocyte Ratios in Detecting Occult Papillary Thyroid Microcarcinomas in Benign Multinodular Goitres |
| 5 | Derya Kocer, 2015 | May the Neutrophil/Lymphocyte Ratio Be a Predictor in the Differentiation of Different Thyroid Disorders? |
| 6 | Satriya Kelana, 2022 | The performance of various laboratory parameters to differentiate follicular thyroid carcinoma and follicular thyroid adenoma |
| 7 | Hakan Bölükbaş, 2020 | Neutrophil to iymphocyte ratio: Does it really differentiate between papillary thyroid carcinomas and multinodular goiter |
| 8 | Burcin Meryem Atak Tel, 2021 | Platelet to lymphocyte ratio in differentiation of benign and malignant thyroid nodules |
| 9 | Hayri Bostan, 2022 | The predictive value of hematologic parameters in the risk of thyroid malignancy in cases with atypia/follicular lesion of undetermined signifcance |
| 10 | Mustafa C Şenoymak, 2024 | Assessment of inflammatory parameters as predictive markers for malignancy in thyroid nodules: a study on the correlation with Bethesda classification |
| 11 | Haider Salim Mihson, 2022 | The Role of Neutrophil to Lymphocyte and Platelet to Lymphocyte Ratios in Diagnosing Thyroid Nodule |
| 12 | Chiara Off, 2021 | Evaluation of LMR, NLR and PLR as predictors of malignancy in indeterminate thyroid nodules |
